# Genome Context as a Predictive Tool for Identifying Regulatory Targets of the TetR Family Transcriptional Regulators

**DOI:** 10.1371/journal.pone.0050562

**Published:** 2012-11-30

**Authors:** Sang Kyun Ahn, Leslie Cuthbertson, Justin R. Nodwell

**Affiliations:** 1 Department of Biochemistry and Biomedical Sciences, McMaster University, Hamilton, Ontario, Canada; 2 M. G. DeGroote Institute for Infectious Disease Research, McMaster University, Hamilton, Ontario, Canada; Baylor College of Medicine, United States of America

## Abstract

TetR family transcriptional regulators (TFRs) are found in most bacteria and archea. Most of the family members that have been investigated to date are repressors of their target genes, and the majority of these, like the well-characterized protein TetR, regulate genes that encode transmembrane efflux pumps. In many cases repression by TFR proteins is reversed through the direct binding of a small-molecule ligand. The number of TFRs in the public database has grown rapidly as a result of genome sequencing and there are now thousands of family members; however virtually nothing is known about the biology and biochemistry they regulate. Generally applicable methods for predicting their regulatory targets would assist efforts to characterize the family. Here, we investigate chromosomal context of 372 TFRs from three *Streptomyces* species. We find that the majority (250 TFRs) are transcribed divergently from one neighboring gene, as is the case for TetR and its target *tetA*. We explore predicted target gene product identity and intergenic separation to see which either correlates with a direct regulatory relationship. While intergenic separation is a critical factor in regulatory prediction the identity of the putative target gene product is not. Our data suggest that those TFRs that are <200 bp from their divergently oriented neighbors are most likely to regulate them. These target genes include membrane proteins (26% of which 22% are probable membrane-associated pumps), enzymes (60%), other proteins such as transcriptional regulators (1%), and proteins having no predictive sequence motifs (13%). In addition to establishing a solid foundation for identifying targets for TFRs of unknown function, our analysis demonstrates a much greater diversity of TFR-regulated biochemical functions.

## Introduction

Bacteria adapt to changes in their environment and metabolism by regulating gene expression. One means of coupling chemical stimuli to appropriate transcriptional responses is to take advantage of ‘one-component systems’ (reviewed in [1]). TetR family transcriptional regulators (TFRs) are widely distributed in bacteria and archea (reviewed in [2]) and they constitute one of the largest groups of one-component transcription factors [3]. TFRs are easily identified through the high sequence conservation in their N-terminal DNA-binding domains [2]; however, their C-terminal domains – which in many of the characterized TFRs interact with small-molecule ligands – are highly variable, suggesting that this family can respond to a diverse range of stimuli.

TetR, one model for this family, is a repressor of *tetA,* which encodes a tetracycline efflux pump (reviewed in [4]). The *tetR* gene is divergently oriented to *tetA*, and the intergenic DNA that separates them contains two 15 bp palindromic operator sequences that are bound by the dimeric TetR to repress transcription initiation from the promoters of both genes [5]. Tetracycline activates *tetA* expression by binding TetR [6] and lowering its affinity for DNA [7]. TetA then exports tetracycline to confer resistance [8].

The majority of characterized TFRs are repressors, though a small number of activators [9], [10], [11] and dual repressor/activators [12], [13] are also known. Like TetR, the majority of the previously studied TFRs regulate genes encoding efflux pumps that confer antibiotic resistance. This includes AcrR in *Escherichia coli* [14], ActR in *Streptomyces coelicolor* [15], [16], NfxB in *Pseudomonas aeruginosa* [17], QacR in *Staphylococcus aureus* [18], and SmeT in *Stenotrophomonas maltophilia* [19]. However TFRs have been implicated in the regulation of other physiological processes including antibiotic biosynthesis [10], the tricarboxylic acid cycle [20], biofilm formation [21], quorum sensing [13], and toxin production [22].

The number of TFRs encoded in genome databases exceeded 20,000 distinct sequences in 2010 [23] and continues to grow. Of this number, only a tiny fraction has been characterized in any detail. Thus, for all but a few TFRs cognate ligands and target genes are unknown. Generally applicable tools for identifying basic elements of the biological roles of TFRs would greatly accelerate our ability to assign functions to this important family of transcriptional regulators.

In this work, we have identified 372 genes encoding TFRs in three streptomycetes – *S. coelicolor, Streptomyces avermitilis*, and *Streptomyces griseus.* We have explored the genome context of these genes and find that most are encoded divergently to a neighboring gene. The TetR paradigm suggests that these are putative target genes. We explored the prediction that these TFRs regulate the divergently encoded neighboring genes and find that this is the case for most or all TFRs where the intergenic separation is less than 200 bp. This is true regardless of the nature of the target gene product. In addition to confirming that the TetR regulatory paradigm holds for a majority of TFRs, our analysis demonstrates a far greater diversity of TFR targets than previously appreciated. While 22% of these proteins control the expression of membrane-associated pumps, the majority of TFRs are predicted to control the expression of targets that encode enzymes.

## Results

### Most TFRs are Divergently Oriented to an Adjacent Gene

We searched the genomes of *S. coelicolor*, *S. griseus*, and *S. avermitilis* for genes encoding putative TFRs and identified 153, 104, and 115 of them, respectively (total of 372 TFRs) based on a high score for the consensus sequence of the protein family PF00440 (TetR_N). Actinomycete chromosomes are linear and share a conserved genetic ‘core’ region and more variable ‘arm’ regions at both ends, containing primarily non-essential species-specific genes including many involved in secondary metabolism [24]. The TFR genes in these streptomycetes are distributed evenly over the chromosomes with a slight enrichment in the ‘core’ relative to the ‘arm’ regions. For example, *S. coelicolor* has 93 TFRs in the ‘core’ (4.9 Mb, approximately 19 TFRs/Mb), 27 TFRs in the left ‘arm’ (1.5 Mb, 18 TFRs/Mb) and 30 TFRs in the right ‘arm’ (2.3 Mb, 13 TFRs/Mb). In addition, *S. coelicolor* contains the SCP1 plasmid (356 kb), which includes three more TFRs.

**Figure 1:**
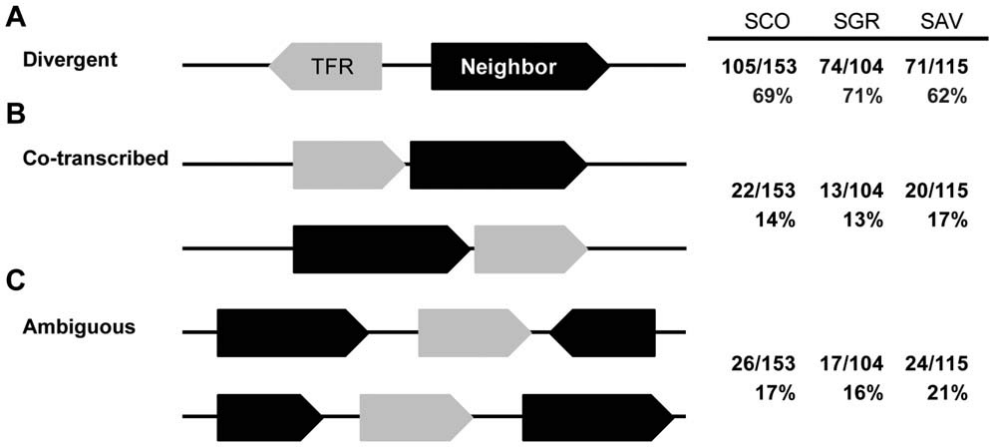
Classification of TFRs according to their relative orientation to the neighboring genes. 372 TFRs in *S. coelicolor* (SCO, 153 TFRs), *S. griseus* (SGR, 104 TFRs), and *S. avermitilis* (SAV, 115 TFRs) were divided into three groups according to their genome context to neighbors. (**A**) 250 TFRs (105 in SCO, 74 in SGR, 71 in SAV) are encoded divergently to their neighbors. Here, a TFR-encoding gene is located on the left side for visualization purpose, but the positions of this gene and its divergent neighbor are interchangeable. (**B**) 55 TFRs (22 in SCO, 13 in SGR, 20 in SAV) are likely co-transcribed with their upstream or downstream genes as the intergenic DNAs separating them are ≤35 bp. (**C**) 67 TFRs (26 in SCO, 17 in SGR, 24 in SAV) show neither of the two aforementioned orientations.

Given the model TetR/TetA regulatory paradigm, we predicted that most of these TFRs regulate the expression of adjacent genes. We examined the genome context of the individual TFRs and divided them into three groups according to their orientation relative to neighboring genes. As shown in Figure 1A, one group is divergently oriented relative to a neighboring gene, like TetR. A second group (Figure 1B) is likely to be co-transcribed with an upstream or downstream neighbor. A small number of TFRs (eight in *S. coelicolor*, four each in *S. griseus* and *S. avermitilis*) have a divergent neighbor on one side and a probable co-transcribed neighbor on the other (included in the first group in Figure 1). The remaining TFRs do not have either of these relationships with the neighbors (Figure 1C). TFRs oriented divergently to their neighboring genes are most common in all three streptomycetes examined and comprise 67% (250 TFRs) of the total TFRs, while 15% (55 TFRs) and 18% (67 TFRs) of the TFRs are in the second and third group, respectively.

We investigated the TFRs of four organisms at various phylogenetic distances from *Streptomyces* – *Mycobacterium tuberculosis* H37Rv (Actinobacteria, Gram-positive and high GC content, 49 TFRs), *Bacillus subtilis* subsp. subtilis str. 168 (Firmicutes, Gram-positive and low GC content, 18 TFRs), *P. aeruginosa* PAO1 (Gammaproteobacteria, Pseudomonadaceae, Gram-negative and high GC content, 40 TFRs), and *E. coli* str. K-12 MG1655 (Gammaproteobacteria, Enterobacteriaceae, Gram-negative and low GC content, 13 TFRs). In correlation with our analysis of the TFRs in the three streptomycetes, the divergent orientation is most frequent in these organisms, although it is less dominant in *B. subtilis* (9 TFRs, 50%) compared to the other three organisms (32 TFRs, or 65%, in *M. tuberculsosis*; 27 TFRs, or 68%, in *P. aeruginosa*; and 10 TFRs, or 77%, in *E. coli*). This analysis suggests that in bacteria, most TFRs will be divergently oriented to their neighbors.

### Variable Features of TFRs and their Divergently Oriented Neighbors

**Figure 2:**
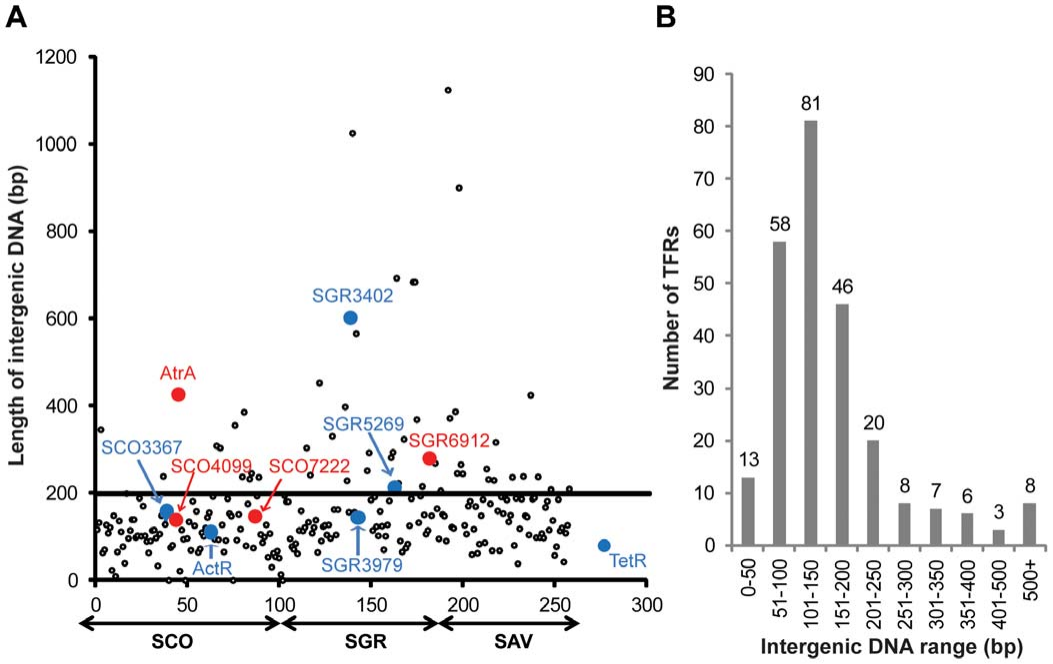
Length of intergenic DNAs between TFRs and their divergent neighbors. (**A**) Each of the 250 TFRs having divergent neighbors in SCO, SGR, and SAV is represented as a dot with the value on y-axis indicating the length of the intergenic sequence between its own gene and divergent gene. On x-axis, the TFRs are placed in the order of their gene annotations along the length of the linear chromosomes (the host streptomycete is stated below). The larger colored dots correspond to the TFRs investigated in this study (see Table 1 and text for details). A model TFR, TetR, is shown on the graph as a reference. Blue dots indicate the TFRs whose divergent neighbors encode putative membrane transporters, while the TFRs represented by red dots are adjacent to genes encoding putative enzymes. (**B**) The TFRs having divergent neighbors are grouped according to the range of their intergenic DNA length.

We investigated the relationship of the 250 TFRs having divergent neighbors from *S. coelicolor*, *S. griseus*, and *S. avermitilis*. First we explored the length of the DNA separating each TFR-encoding gene from its putative target (Table S1, note that the separation in bp is reported relatively to the genes’ translational start sites as the transcriptional start sites are unknown in the overwhelming majority of cases). As shown in Figure 2A, the length of this DNA varies from 0 bp to 1123 bp. However, most intergenic regions (198 of 250, or 79%) are ≤200 bp (Figure 2B). A similar pattern was observed in *P. aeruginosa* and *M. tuberculosis* with 74% (20 TFRs) and 75% (24 TFRs) of their respective TFRs having divergent neighbors less than 200 bp away from the adjacent open reading frames. On the other hand, the intergenic regions in this size range are less frequent in *B. subtilis* (5 TFRs, 56%) and *E. coli* (5 TFRs, 50%) although this may be exaggerated by the smaller sample size in these organisms.

**Table 1:** Nine TFRs of interest and their divergent neighbors.

TFR of interest	Predicted divergent gene product	Length of intergenic DNA
*TFRs whose divergent genes encode putative transporters*		
ActR (SCO5082)	ActA (SCO5083, MFS)	110 bp
SGR3979	SGR3977/SGR3978 (ABC)	144 bp
SCO3367	SCO3366 (MFS)	158 bp
SGR5269	SGR5270 (MFS)	212 bp
SGR3402	SGR3403 (MFS)	601 bp
*TFRs whose divergent genes encode putative enzymes*		
SCO4099	SCO4098 (Acyltransferase, EC 2)	139 bp
SCO7222	SCO7223 (Monooxygenase, EC 1)	146 bp
SGR6912	SGR6911 (Glycosyl hydrolase, EC 3)	280 bp
AtrA (SCO4118)	SCO4119 (NADH dehydrogenase, EC 1)	425 bp

**Figure 3:**
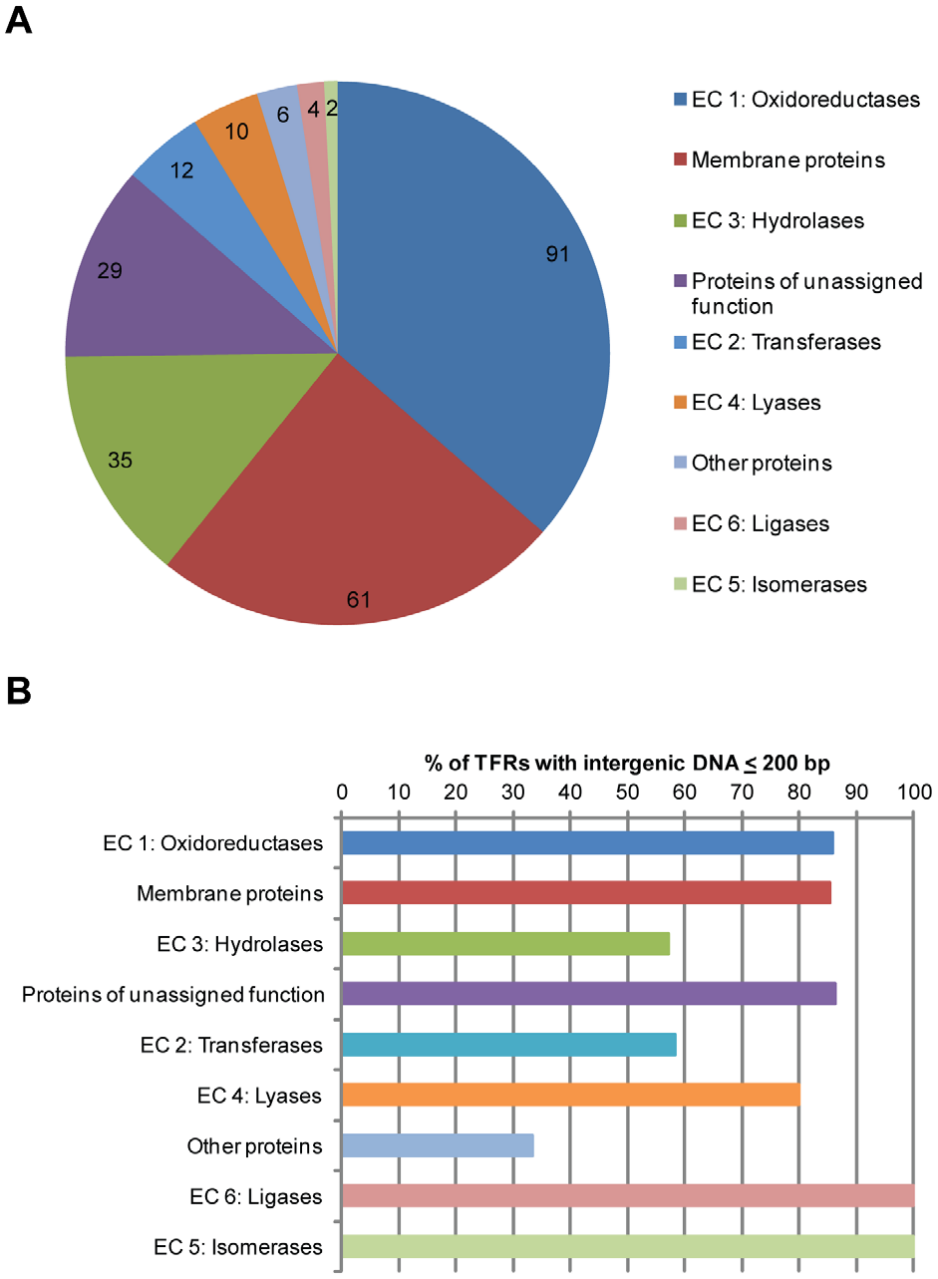
Diverse product types encoded by the divergent neighboring genes. (**A**) The number of TFRs adjacent to each type of divergent gene products – sorted by enzymes in six groups (EC 1 to EC 6), membrane proteins, other proteins (e.g. transcriptional regulators), and proteins of unassigned function. (**B**) TFRs were grouped according to their divergent gene type and the percentage of TFRs having intergenic DNAs ≤200 bp for each group is shown.

We next analyzed the protein products encoded by the divergent neighboring genes using protein BLAST and Conserved Domain Search (CD-Search, discussed in [25]) (Table S1). As shown in Figure 3A, the predicted gene products include putative enzymes (154 of 250, or 62%), membrane proteins (61, or 24%), and other proteins such as transcriptional regulators (6, or 2%). The function of 29, or 12%, of the putative targets could not be predicted as they lack any known motif and/or have no BLAST hit with proteins of known function.

The predicted enzymes were further divided based on two criteria: the Enzyme Commission (EC) number to indicate the type of the chemical reactions they are predicted to catalyze [26] as well as any conserved domain they possess. As demonstrated in Figure 3A and Table S2, our analysis revealed that the 154 putative enzymes include members in all six known EC groups (i.e. EC 1 to EC 6). For example, 91 of the 154 putative enzymes are predicted to be oxidoreductases (EC 1). 51 of these have a conserved sequence of the Rossmann fold (NADB_Rossmann, cl09931, in Table S2), which is characterized by the Gx_1–2_GxxG motif [27] and known to be one of the three most common folds in the Protein Data Bank [28]. A large number of proteins containing the Rossmann fold bind to nucleotide cofactors such as FAD and NAD(P) and function as oxidoreductases such as lactate dehydrogenases and flavodoxins [29]. On the other hand, eight proteins are grouped in the acyl-CoA dehydrogenase superfamily (ACAD, cl09933, in Table S2), known to be involved in a broad spectrum of primary and secondary metabolic processes such as the β-oxidation of fatty acids [30] and antibiotic biosynthesis [31].

Among the membrane proteins encoded by the putative target genes, 84% (51 of 61) are predicted to be transporters while the remainders contain putative transmembrane segments but lack any other predictive sequence motif (Table S2). While 26 of the transporters are predicted to belong to the major facilitator superfamily (MFS), the others belong to families such as the ATP-binding cassette (ABC) or resistance-nodulation-division (RND) transporter families.

Certain gene types such as EC 1 oxidoreductases (36%) and membrane proteins (24%) were found more frequently than others (e.g. EC 6 ligases, 2%, and EC 5 isomerases, 1%) (Figure 3A). There was no obvious correlation between the length of the intergenic DNA and the type of divergent gene product (Figure 3B).

While two of the best characterized TFRs, TetR and QacR, are divergently oriented to target genes that encode efflux pumps [4], [18], our analysis suggests that there is a much greater diversity in the possible targets regulated by TFRs and most of these genes do not encode export proteins.

### In vitro Analysis of Selected TFRs having Divergent Neighboring Genes

To determine whether the length of the intergenic DNA or the putative function of the neighboring gene correlates with regulation by an adjacent TFR, we selected eight previously uncharacterized TFRs from *S. coelicolor* and *S. griseus* for molecular genetic analysis (Figure 2A and Table 1). We chose TFRs divergent to putative transporters (three MFS and one ABC-type transporters) or enzymes (two EC 1 oxidoreductases, one EC 2 transferase, and one EC 3 hydrolase) with intergenic DNAs of varying lengths (139 bp to 601 bp). In addition, ActR (SCO5082) from *S. coelicolor* was used as a well-characterized control [15], [16], [32]. The coding sequences of these proteins were amplified, subcloned, and expressed in *E. coli* such that they could be purified via His_6_-tags.

**Figure 4:**
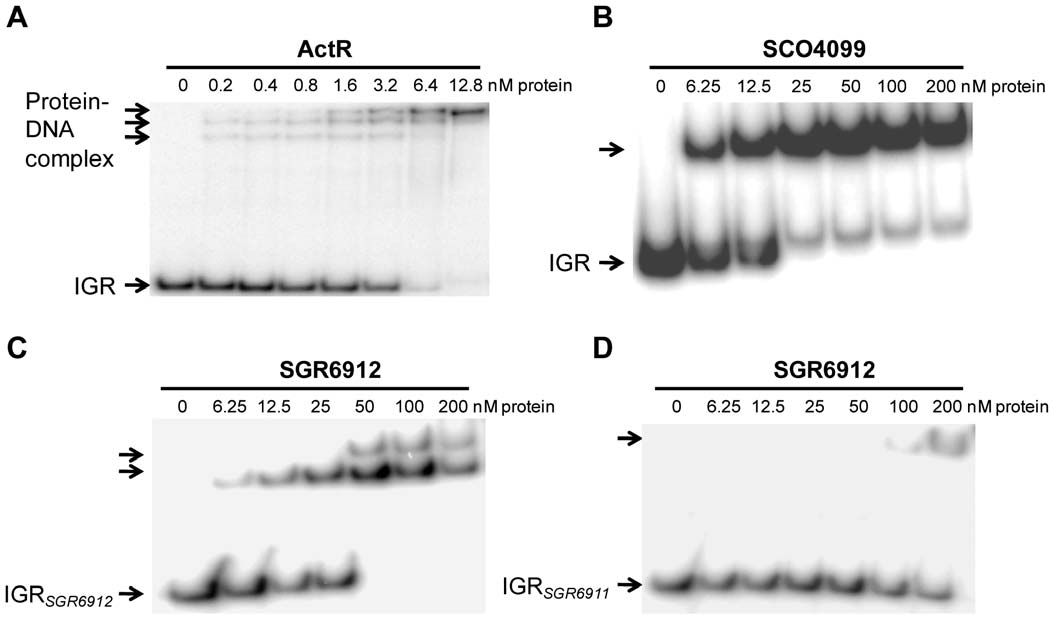
ActR, SCO4099, and SGR6912 bind the intergenic DNAs between their own genes and divergent neighbors. EMSAs showing the interactions between (**A**) ActR and the entire sequence of the *actR*/*actA* intergenic region (IGR); (**B**) SCO4099 and the entire sequence of the *SCO4098*/*SCO4099* IGR; (**C**) SGR6912 and the IGR*_6912_* probe that contains the 200 bp sequence upstream of the *SGR6912* translational start (partial *SGR6911*/*SGR6912* intergenic sequence); and (**D**) SGR6912 and the IGR*_6911_* probe that contains the 200 bp sequence upstream of the *SGR6911* translational start site (partial *SGR6911*/*SGR6912* intergenic sequence). The indicated concentrations of a TFR were incubated with a ^32^P-labeled DNA fragment containing the entire or partial intergenic sequence between the TFR-encoding gene and its divergent neighboring gene. Unbound DNA fragment is indicated by the bottom arrow (IGR, IGR*_6912_*, or IGR*_6911_*), while the shifts representing protein-DNA complexes are indicated by the upper arrows.

**Figure 5:**
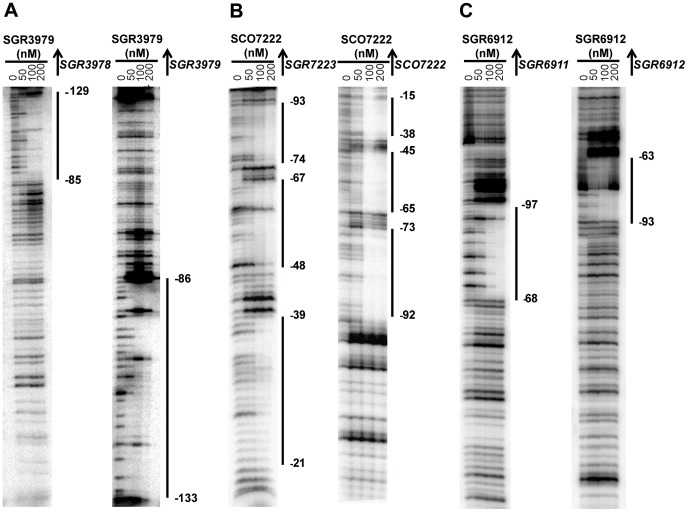
SGR3979, SCO7222, and SGR6912 display different DNA protection patterns on the cognate intergenic DNAs. DNaseI footprinting assays showing the protection patterns of (**A**) SGR3979 on the entire *SGR3978*/*SGR3979* intergenic sequence; (**B**) SCO7222 on the entire *SCO7222*/*SCO7223* intergenic sequence; and (**C**) SGR6912 on the IGR*_6912_* sequence containing the partial *SGR6911*/*SGR6912* intergenic region (same as Figure 4C). In the presence of the indicated concentrations of a TFR, a DNA fragment containing the entire or partial intergenic sequence between the TFR-encoding gene and its divergent neighboring gene was exposed to DNaseI. For the left gel for each TFR, the primer that was extended toward its divergent neighboring gene was labeled at 5′-end to prepare the probe, while the other primer extended toward its own gene was labeled to prepare the probe for the right gel. The regions protected by the TFRs are indicated by solid vertical lines. The numbers beside each line indicate the start and end positions of the protected region relative to the translational start site of the TFR-encoding gene.

We conducted electrophoretic mobility shift assays (EMSAs) to determine whether the nine TFRs bound their respective intergenic DNAs. As shown in Figure 4A and B, ActR (intergenic DNA = 110 bp) and SCO4099 (139 bp) formed tight complexes with their cognate intergenic sequences. Although the numbers of protein-DNA complexes – consistent with the number of discrete binding sites – detected for ActR (three complexes) and SCO4099 (one complex) were different, the mobility shifts were observed at the protein concentrations as low as 0.2 nM and 6.25 nM, respectively. Similar observations were made with SGR3979 (144 bp), SCO7222 (146 bp), SCO3367 (158 bp), and SGR5269 (212 bp) (Figure S1A), all of which have intergenic sequences close to or smaller than 200 bp. We used competition assays to confirm that the interactions of SCO4099 and SGR3979 with their cognate intergenic regions were specific (Figure S2). We have not conducted competition assays with the other TFRs under investigation in this work as the footprinting data confirm that each protein interacts with a discrete and distinct recognition sequence (see Figure 5 and Figure S3).

The intergenic sequences of SGR6912 (280 bp), AtrA (SCO4118, 425 bp), and SGR3402 (601 bp) are much longer than 200 bp so they were divided into multiple, overlapping probes for *in vitro* assays (two probes for SGR6912, three for AtrA, and four for SGR3402). Similar to our observations with the TFRs with shorter intergenic sequences, both SGR6912 (Figure 4C and D) and AtrA (Figure S1B) bound their cognate intergenic sequence fragments. SGR6912 clearly bound more tightly to the probe closer to its own gene (IGR*_SGR6912_*, shift observed at 6.25 nM, Figure 4C) than to the other probe closer to its divergent neighboring gene *SGR6911* (IGR*_SGR6911_*, shift at 100 nM, Figure 4D). Similarly, a very high concentration of AtrA, 100 nM, was required for the formation of a detectable complex with the DNA probe closest to the divergent *SCO4119* gene (Figure S1B). In contrast, the two probes closer to the *atrA* gene itself formed tight complexes with 12.5 nM or 25 nM of the protein. Finally, SGR3402 – with the longest intergenic sequence – did not interact with any of the four probes even at the highest protein concentration tested (400 nM, Figure S1C). While it is possible that SGR3402 binds within the open reading frame(s) of *SGR3402* and/or *SGR3403*, these results imply that SGR3402 does not regulate the *SGR3403* expression.

**Figure 6:**
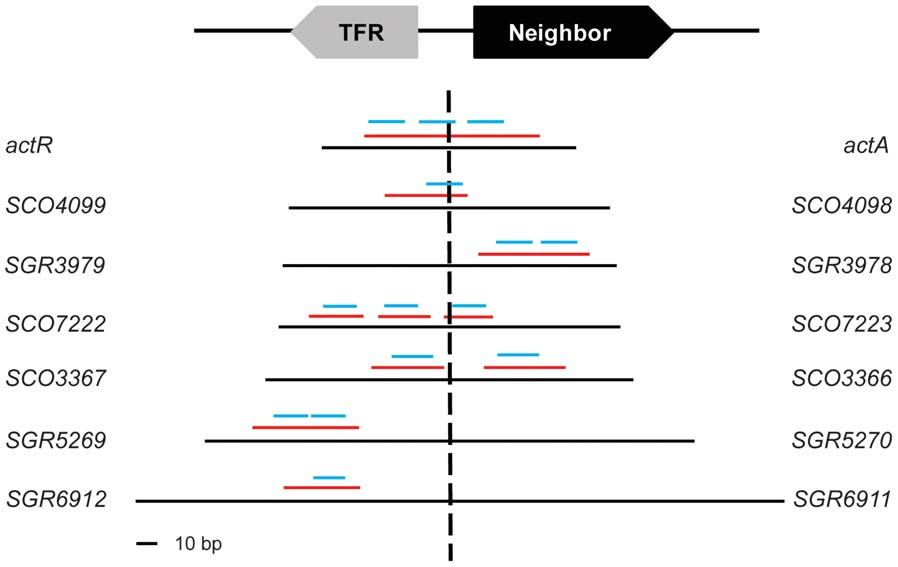
Seven TFRs bind different regions in the intergenic DNAs relative to their divergent neighbors. Solid black horizontal lines represent the intergenic DNAs, while red horizontal lines indicate the regions protected by the TFRs, on one or both strands of the DNAs. Putative operators of the TFRs were identified through sequence analysis of the protected regions, and their positions are indicated by blue horizontal lines. These lines are oriented such that all of the TFR-encoding genes are located on the left side while their divergent neighboring genes are located on the right side. Dashed vertical line represents the center of the intergenic DNAs.

We mapped the binding sites of the eight TFRs that bound the cognate intergenic sequences through DNase I footprint assays on both DNA strands, observing protected regions ranging from 15–48 bp (Figure 5 and Figure S3). Importantly, these protected regions were observed at differing locations relative to their neighboring genes (summarized in Figure 6). For example, SGR3979 protected a single region (Figure 5A) located close to the divergent gene *SGR3978*, while SCO7222 bound three discrete regions (Figure 5B). Two of the SCO7222 binding sites are located closer to its own gene, while the remaining site is closer to *SCO7223*. For SGR6912, the assays were conducted with both probes of the IGR*_SGR6911_* and IGR*_SGR6912_* sequences. Protection by SGR6912 was only observed with the IGR*_SGR6912_* probe (Figure 5C), indicating that the operators of this TFR are positioned more proximal to *SGR6912*.

SGR5269 behaved similarly to SGR6912 and only bound a single region (Figure S3A) adjacent to its own gene, while SCO3367 had two binding sites (Figure S3B) – one closer to *SCO3367* and the other one closer to *SCO3366*. Both ActR and SCO4099 bound near or at the centre of their respective intergenic sequences although SCO4099 protected a much smaller region than ActR (Figure S3C and D). No footprint was obtained with AtrA (data not shown) although the previous EMSA experiments indicated that this TFR can bind its intergenic sequence (Figure S1B). Of note, most of the DNA protection patterns exhibited by these TFRs, except SCO3367, are dissimilar to what has been reported with TetR, which binds two distinct regions containing the *tetR*-proximal and *tetA*-proximal operators in order to regulate both genes [5].

Candidate operator sequences were identified within the regions protected by the seven TFRs with successful footprints, and they correlated well with the numbers of protein-DNA complexes we had observed by EMSA (Table 2). For example, we have previously identified three perfect repeats of the consensus TGGAACGNCGTTCCA in the *SCO7222*/*SCO7223* intergenic region and predicted them to be operators for the *SCO7223* gene promoter sequence [32]. All three repeats (Table 2) were found within the regions bound by SCO7222 (Figure 6), consistent with the three protein-DNA complexes this TFR formed with the intergenic sequence (Figure S1A). Similarly, the region protected by ActR had three weaker palindromes (Table 2 and Figure 6) containing a previously identified direct or inverted sequence of CCACCGTT [16], [32], correlating well with the three shifts detected (Figure 4A).

**Table 2:** Putative operator sequences of the seven TFRs with successful footprints.

TFR	Number of shifts observed by EMSA	Putative operator sequence^a^
ActR	3	G*AACGG*GCCA*CCGTT*T
		CGC*GA* C*C*ACC*G*T*T* *C*CAT
		A*GAACG* GTGGT*CGTTC*G
SGR3979	2	*TGCGTAA* TGC *TTACGCA*
		C*GCGTATG* G*C* *ATACGC* A
SCO3367	2	*ACT* T*G* A*CG*CC*CG* G*C*T*AGT*
		*ACTTGCCG * GG*CGGCAAGT*
SGR5269	2	*TTGC* G*CA* G*TG*G*GCAA*
		T*TGC* C*CA* G*TG*T*GCA*T
SCO4099	1	*CAC* CT*GT*CGC*AC*TA*GTG*
SCO7222	3	*TGGAACG* T*CGTTCCA*
		*TGGAACG* A*CGTTCCA*
		*TGGAACG* C*CGTTCCA*
SGR6912	2	*ACTAA* CCAC*TTAGT*

a The palindromic nucleotides are italicized, while the repeated nucleotides are underlined.

The only exception was SGR6912, for which only one palindrome (Table 2) was identified within the protected region (Figure 6) in contrast to the two shifts detected with the IGR*_SGR6912_* probe by EMSA (Figure 4C). Interestingly, no effect was observed when this sequence was used in competition with the IGR*_SGR6912_* probe (data not shown), suggesting that this sequence does not contain all the nucleotides required for efficiently interacting with SGR6912. The actual operator might therefore consist of an extended sequence (at one or both ends) capable of binding two protein dimers, possibly in a cooperative manner. Of note, only a part of this putative operator sequence is conserved in the IGR*_SGR6911_* probe (missing the first three nucleotides of the putative operator shown in Table 2), within the region it overlaps with IGR*_SGR6912_*. This might explain the considerably lower affinity SGR6912 has for this probe (Figure 4D) compared to the IGR*_SGR6912_* probe (Figure 4C). Therefore, the lack of protection by SGR6912 on IGR*_SGR6911_* observed in footprinting assays is likely due to the weakness of this interaction and/or the fact that the putative SGR6912 binding site is interrupted at end of the IGR*_SGR6911_* probe (where optimal resolution of protected region was not possible).

More importantly, the TFRs having shorter intergenic sequences (i.e. ActR, SCO4099, SGR3979, SCO7222, and SCO3367) tended to bind the operators located proximal to both the TFR gene and the putative target, or to bind proximally to the putative target (Figure 6). In contrast, it is evident that the two TFRs with larger intergenic sequences (i.e. SGR5269 and SGR6912) bind the operator sequences that are distal from the divergent genes (Figure 6). These results, combined with the observation that SGR3402 did not interact with its intergenic sequence (Figure S1C), suggest that the length of an intergenic sequence might be predictive of a regulatory relationship between a TFR and a divergently oriented gene.

### Regulatory Activity of the TFRs on their Divergent Neighbors

**Figure 7:**
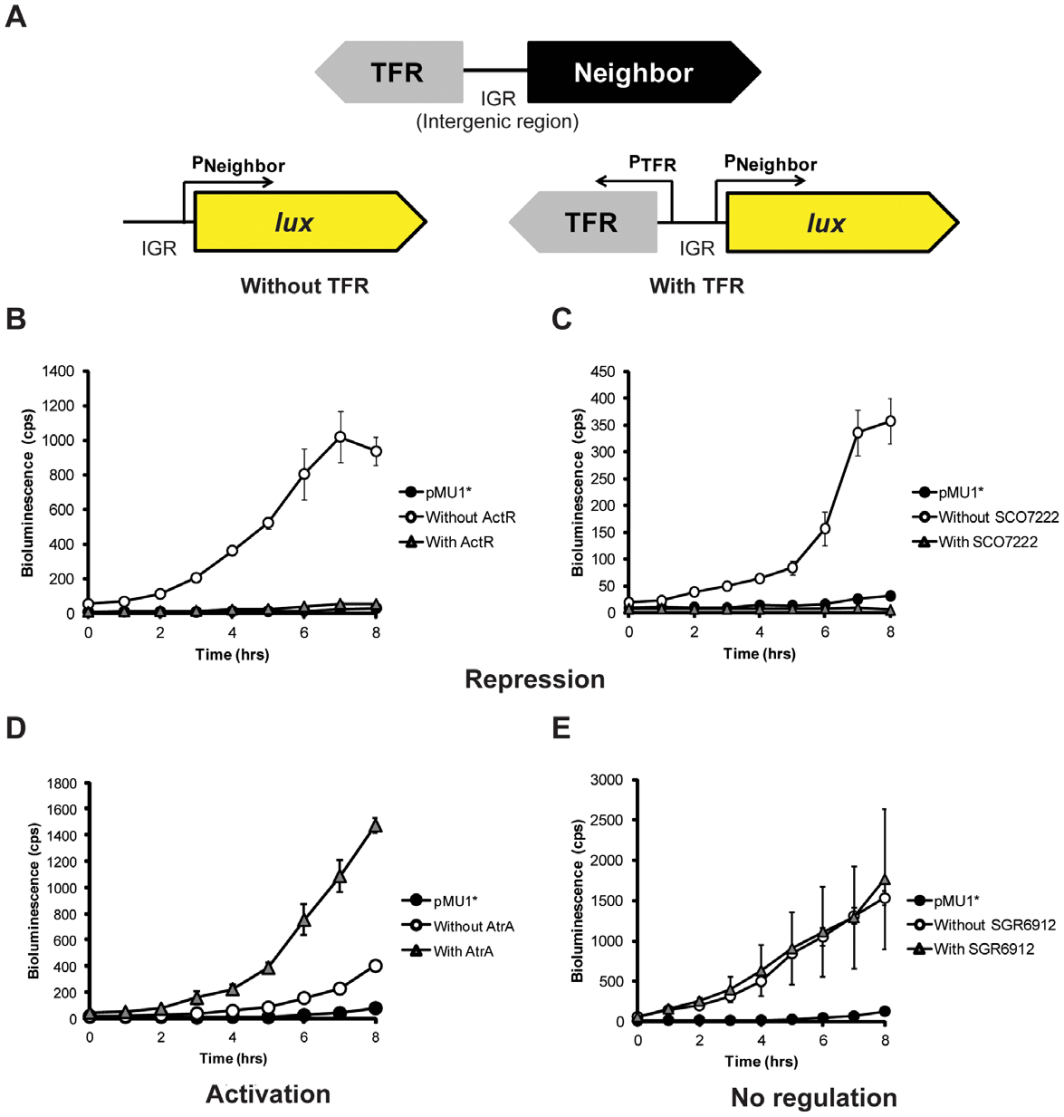
ActR, SCO7222, AtrA, and SGR6912 possess different regulatory effects on the divergent neighbors. (**A**) Two reporter plasmids were constructed for each TFR. For these plasmids, expression of the *lux* operon is driven by the promoter of the divergently transcribed neighboring gene in the absence (Without TFR) or in the presence (With TFR) of the TFR-encoding gene in *cis*. These two plasmids were introduced separately into a heterologous *Streptomyces* host for comparing their bioluminescence production as a function of time. Average bioluminescence values, measured in counts per second (cps), as well as +/− standard deviation of the values were obtained from at least three independent readings. Compared to the “Without TFR” constructs, three different outcomes were displayed when the cognate TFRs were expressed in *cis*. (**B**) Negative effect on luminescence by ActR. (**C**) Negative effect on luminescence by SCO7222. (**D**) Positive effect on luminescence by AtrA. (**E**) No effect on luminescence by SGR6912.

To biologically assess the regulatory activity of the nine TFRs on their neighboring genes, we used a *luxCDABE* operon [33] to create transcriptional reporters. Two reporter plasmids were constructed for each TFR: a “promoter only” construct where expression of the *lux* operon is driven by the promoter of the divergently transcribed neighboring gene (“without TFR” in Figure 7A) and a second reporter where the TFR gene was included in *cis* (“with TFR” in Figure 7A).

To avoid interference from chromosomally encoded TFRs acting in *trans*, we introduced each of the reporters into a sequenced heterologous host. To choose an appropriate host for each reporter we used protein BLAST to identify a streptomycete that did not possess any TFR with 40% or greater protein sequence identity (Table 3). For each TFR, we introduced the two reporter constructs separately into a selected host and monitored growth and bioluminescence as a function of time. The only exception was AtrA, which occurs in all streptomycetes (unpublished data), and its reporters were introduced into the natural host *S. coelicolor*.

**Table 3:** Selected heterologous *Streptomyces* host for each TFR in the bioluminescence assays.

TFR	Host	Top BLAST hit^a^
ActR	*S. venezuelae* ATCC 10712	SVEN3777–27(45)
SGR3979	*S. coelicolor* M145	SCO4358–39(52)
SCO3367	*S. albus* J1074	SSHG_05469–26(46)
SGR5269	*S. coelicolor* M145	SCO2374–36(50)
SGR3402	*S. coelicolor* M145	None
SCO4099	*S. sviceus* ATCC 29083	SSEG_10996–38(52)
SCO7222	*S. venezuelae* ATCC 10712	SVEN6489–38(53)
SGR6912	*S. coelicolor* M145	None
AtrA	*S. coelicolor* M145	AtrA –100(100)

a Top BLAST hits with at least 75% query coverage are indicated. The amino acid sequence identity and similarity (in the bracket) are shown.

As shown in Figure 7B to E and Figure S4, luminescence from the “promoter only” constructs was greater than that of the promoterless vector control (3-fold to 197-fold at t = 8 h) while growth rate was unchanged (data not shown). The promoters of the putative target genes were therefore all active in the heterologous species.

Compared to the “promoter only” constructs, three different outcomes were obtained when the cognate TFRs were expressed in *cis*. As expected, in the presence of ActR, luminescence from P*_actAB_* was reduced 23-fold at t = 8 h (Figure 7B). This is consistent with the previous studies showing that ActR represses the *actAB* promoter [15], [16] and it validates our reporter system. Similar results were observed when SCO7222 (72-fold reduction, Figure 7C), SGR3979 (47-fold reduction, Figure S4A), SCO3367 (83-fold reduction, Figure S4B), and SCO4099 (33-fold reduction, Figure S4C) were expressed in *cis*.

In contrast, AtrA appeared to enhance the *lux* expression by 4-fold compared to the “promoter only” construct (Figure 7D). These data suggest a role for AtrA in activating expression of its divergent neighboring gene *SCO4119* (encoding a putative NADH dehydrogenase), and it is consistent with the previously documented effect of AtrA as a transcriptional activator. In previous work this protein was shown to positively regulate the expression of *act*II-ORF4, which in turn activates the expression of genes involved in the biosynthesis of the actinorhodin [10].

On the other hand, expression of SGR5269, SGR6912, and SGR3402 had no effect on luminescence as compared to their cognate “promoter only” constructs (Figure 7E, Figure S4D and E). One possibility for this observation is lack of the TFR expression in the heterologous host under the conditions tested. To rule out this possibility, we constructed reporters where *lux* expression is driven by the promoter of the TFR itself. Luminescence from each of these reporters was above that of the vector control (data not shown). It could be speculated that these TFRs require ligands or co-regulator proteins to elicit activity and that these are not present; however, this is unlikely for SGR5269 and SGR6912 as they tightly bound their target DNAs *in vitro* without any addition of co-factor (Figure 4C and Figure S1A). Another possibility is that ligands of these TFRs are present in the selected host and they prevent the TFRs from binding the operators although this is unlikely for SGR3402 as it did not bind DNA *in vitro* without the presence of any added ligand (Figure S1C). We have not ruled out these possibilities, however, the most likely explanation is that SGR5269, SGR6912, and SGR3402 do not regulate their divergent neighboring genes – *SGR5270*, *SGR6911*, and *SGR3403*, respectively. Therefore, the interactions of SGR5269 and SGR6912 with their intergenic DNA sequences *in vitro* likely indicate that these TFRs are autoregulatory and do not act as repressors or activators of the promoters of their divergent neighbors.

These reporter assays underscore the correlation between the length of the intergenic sequence and the regulatory activity of TFRs observed in our *in vitro* data (Figure 4, Figure 5, Figure S1, and Figure S3). All five of the TFRs (ActR, SCO4099, SGR3979, SCO7222, and SCO3367) with the intergenic sequences <200 bp repressed the promoters of their divergently oriented neighboring genes, like TetR. On the other hand, three (SGR5269, SGR6912, and SGR3402) of the four TFRs with the intergenic sequences >200 bp did not display any regulatory activity on their divergently transcribed neighboring genes while the fourth TFR, AtrA, activated expression.

No correlation was observed between the biochemical activity of the divergent gene product and the regulatory role of the adjacent TFR. ActR, SGR3979, and SCO3367 control expression of the genes encoding putative export pumps while SCO4099, SCO7222, and AtrA control expression of the genes encoding putative enzymes. These data suggest that the physiological processes under the regulation of TFRs include a great diversity of enzymatic functions; in fact, export proteins constitute a minority of target gene products.

## Discussion

The majority of the genes encoding TFRs (67%) in *S. coelicolor*, *S. griseus*, and *S. avermitilis* are transcribed divergently to an adjacent gene. The lengths of the intergenic DNA sequences separating the two genes are highly variable however, in most cases the separation is less than 200 bp. Our data suggest that those TFRs having intergenic DNAs <200 bp are, in most or all cases, likely to be repressors of the divergent genes. As evidence for this, we have confirmed that ActR is a repressor of *actAB* and also demonstrated repression of *SCO4098*, *SGR3978*, *SCO7223*, and *SCO3366* by their cognate divergent TFRs – SCO4099, SGR3979, SCO7222, and SCO3367, respectively. Consistent with our analysis, many previously characterized TFRs obey this ‘200 bp’ rule, including EbrS (intergenic DNA = 65 bp), EthR (75 bp), TetR (81 bp), SimR (138 bp), DesT (158 bp), QacR (177 bp), XdhR (188 bp), and LanK (190 bp) [4], [18], [34], [35], [36], [37], [38], [39]. The prediction that such TFRs with intergenic sequences <200 bp will regulate adjacent genes is important because it means that at least one transcriptional target gene can be identified for more than half of all TFRs in the public databases, encompassing at least 25,000 distinct genes (unpublished data).

The regulatory prediction is less reliable for TFRs that are separated from divergent neighboring genes by >200 bp; however, it is worth pointing out that our data do not rule out a classical, TetR-like regulatory relationship for these proteins and indeed, exceptions are known. For example, AtuR in *P. aeruginosa*, BpeR in *Burkholderia pseudomallei*, and Mce3R in *M. tuberculosis* are all TetR-like repressors of divergent neighbors where the intergenic sequences are 280 bp, 409 bp, and 898 bp, respectively [40], [41], [42].

Surprisingly, while most previously characterized TFRs control the expression of export pumps, we find that most of the divergent genes encode putative enzymes: membrane-associated export proteins such as MFS (e.g. ActA and SCO3367) and ABC pumps (e.g. SGR3978) constitute less than 25% of the divergent gene product that obey the ‘200 bp’ rule. Importantly, the TFRs are in most or all cases repressors of the divergent enzyme-encoding genes. The variety of protein products of these genes is enormous and encompasses all known classifications of enzymes such as EC 1 oxidoreductases (e.g. SCO7223) and EC 2 transferases (e.g. SCO4098). It is likely that some of these enzymes are involved in resistance mechanisms for antibiotics or other toxic molecules; however, we suggest that in many cases the biological roles are metabolic in nature. Indeed, of the predicted targets in Table S2, the ‘knowns’ have predicted catalytic mechanisms, but their biochemical and biological roles are completely unknown.

An emerging paradigm suggests that in many cases the small-molecule ligands of TFRs are related or identical to the substrate of the target gene product. Thus, identifying ligands for TFRs of unknown function promises to provide important biochemical and biological insights into these target genes. This idea has led us to create a relational framework, using phylogenetic methods, which describes and organizes the TFR sequence diversity that exists in the current genome database (Cuthbertson, Ahn and Nodwell, manuscript submitted). Our evidence suggests that this framework provides reliable predictions concerning the ligands for hundreds of TFRs based on their sequence homology. Therefore, the combined use of the predictive tools that we have developed for identifying target genes and ligands for TFRs will provide considerable benefit in understanding the biological roles of this important family of transcriptional regulators.

## Materials and Methods

### Genomic and Bioinformatic Analysis of TFRs

TFRs were identified using protein BLAST (blast.ncbi.nlm.nih.gov) with the consensus sequence for Hidden Markov Model (HMM) Pfam PF00440 (TetR_N). The genome context of individual TFRs was analyzed at StrepDB (streptomyces.org.uk) and National Center for Biotechnology Information (NCBI, www.ncbi.nlm.nih.gov), and each TFR was placed in three groups depending on their orientation to neighboring genes. TFRs divergently oriented to their immediate neighboring genes – regardless of the length of intergenic sequences between them – were placed in the first group. The second group contains TFRs that are predicted to be co-transcribed with their upstream and/or downstream genes when separated 35 bp or less, while the members in the last group lack the aforementioned relationships with the adjacent genes. The protein products of the divergent neighboring genes were analyzed using protein BLAST as well as NCBI CD-Search to predict their functions.

### Bacterial Strains, Plasmids, and Culture Conditions

Bacterial strains and plasmids used in this study are described in Table 4 and Table S3, respectively. *E. coli* cultures were grown as previously described [43], using Luria broth (LB) or LB agar medium containing the appropriate antibiotics when required. *Streptomyces* cultures were grown as previously described [44] using MS, R2YE, R5, and MYMTap [36] media.

**Table 4:** Strains used in this work.

Strain	Description	Reference
*Escherichia coli*		
BL21(DE3)	F^−^ *dcm ompT hsdS*(r_B_^−^ m_B_^−^) *gal met* λ(DE3)	Novagen
Top10	F- *mcrA* Δ(*mrr*-*hsdRMS*-*mcrBC*) ψ80*lacZ*ΔM15 *nupG recA1 araD*139 Δ(*ara*-*leu*)7697 *galE*15 *galK*16 *rpsL*(Str^R^) *endA1* λ^−^	Invitrogen
ET12567/pUZ8002	ET12567 containing helper plasmid pUZ8002	[44]
*Streptomyces*		
*S. coelicolor*	M145 prototroph, SCP1- SCP2-	The John Innes Centre
*S. venezuelae*	ATCC 10712 prototroph	The John Innes Centre
*S. sviceus*	ATCC 29083 prototroph	Broad Institute
*S. albus*	J1074 prototroph	Broad Institute

### Procedures for DNA Manipulation

Standard procedures were used for plasmid isolation, manipulation, and analysis [43]. Oligonucleotide primers were obtained from the Institute for Molecular Biology and Biotechnology (MOBIX) facility at McMaster University or from Sigma-Aldrich. Polymerase chain reactions (PCR) were carried out using Vent DNA polymerase (New England Biolabs). DNA sequencing was carried out by the MOBIX facility to select/isolate the appropriate PCR products.

### Expression and Purification of His_6_-tagged TFRs

Previously prepared pET28a-ActR [16] and pTO7222 [32] were used to express and purify N-terminal His_6_-tagged ActR and SCO7222, respectively from *E. coli*. Similarly, *S. coelicolor* and *S. griseus* chromosomal DNAs were used as templates to PCR amplify the DNA fragments containing the *SCO3367*, *SCO4099, atrA* (*SCO4118*), *SGR3402, SGR3979, SGR5269,* and *SGR6912* open reading frames which were introduced separately into pET28a, giving pET28a-SCO3367, pET28a-SCO4099, pET28a-AtrA, pET28a-SGR3402, pET28a-SGR3979, pET28a-SGR5269, or pET28a-SGR6912, respectively (Table S3).

*E. coli* BL21(DE3) cultures containing individual vectors were grown at 37°C to an OD_600_ of 0.4–0.6 and TFR expression was induced through addition of 1 mM isopropyl β-D-thiogalactopyranoside for 3 to 5 hours at 37°C. Cells were collected by centrifugation at 2,700×g for 15 min at 4°C in the Sorvall SLA-3000 rotor and lysed using the BugBuster reagent (Novagen). The lysate was cleared by centrifugation at 17,200×g for 30 min at 4°C in the Sorvall SS-34 rotor and filtered through a 0.45 µm filter to remove smaller debris and insoluble protein. 4 mL of QIAGEN Ni-NTA agarose solution was added to the filtered lysate and the mixture was allowed to incubate for 1 h at 4°C with gentle shaking. The column was washed with buffer A (50 mM Tris, pH 7.9, 0.5 M NaCl, 20 mM imidazole) and eluted in buffer B (50 mM Tris, pH 7.9, 0.5 M NaCl, 1 M imidazole). Elution fractions were monitored by SDS-PAGE. Fractions containing a TFR were pooled and exchanged into buffer C (20 mM Tris, pH 7.9, 0.5 M NaCl, 20% v/v glycerol). The desalted protein was concentrated using an Amicon Ultra Centrifugal Filter (10,000 MWCO; Millipore).

### EMSAs

*S. coelicolor* and *S. griseus* chromosomal DNA templates were used in PCR reactions to isolate double-stranded DNA fragments containing the intergenic sequences – between *actR* (*SCO5082*) and *actA* (*SCO5083*); *SCO7222* and *SCO7223*; *SCO3366* and *SCO3367*; *SGR3978* and *SGR3979*; *SGR5269* and *SGR5270* – which served as the probes for ActR, SCO7222, SCO3367, SGR3979, and SGR5269, respectively in the assays. The probes for AtrA, SGR3402, and SGR6912 were prepared by obtaining the DNA fragments (148 bp to 200 bp in lengths) containing different regions within their intergenic sequences – between *atrA* (*SCO4118*) and *SCO4119*; *SGR3402* and *SGR3403*; *SGR6911* and *SGR6912* respectively – with partially overlapped ends. The DNA fragments were 5′-end labeled using [γ-^32^P] ATP (PerkinElmer) and T4 polynucleotide kinase (New England Biolabs).

A labeled probe (1 ng), varying amounts of a purified protein, and 90 ng of salmon sperm DNA (Sigma-Aldrich) were used in 15 µl reactions containing 1x EMSA reaction buffer (10 mM Tris-Cl (pH 7.8), 150 mM NaCl, 2 mM DTT and 10% glycerol). Reactions were incubated at 30°C for 10 minutes and were fractionated on 12% non-denaturing polyacrylamide gels containing 1.5% glycerol. The gels were exposed using a phosphor screen (Amersham) and bands were detected using a PhosphorImager (Molecular Dynamics).

### DNase I Footprinting Assays

The same pairs of primers to amplify the intergenic sequences in the previous EMSAs were used for DNase I footprinting. The probes in the assays were prepared by PCR using one unlabeled primer and one 5′-end labeled primer (using [γ-^32^P] ATP and T4 polynucleotide kinase). 150,000 cpm of a labeled DNA probe, varying amounts of a purified protein, and 90 ng of salmon sperm DNA (Sigma-Aldrich) were used in 40 µl reactions containing 1x EMSA reaction buffer. After the reactions were incubated at 30°C for 10 minutes, 10 µl DNase I solution (1 U in 10 mM CaCl_2_) was added. The incubation was continued for 60 seconds at room temperature and reactions were stopped by adding 140 µl DNase I stop solution (200 mM NaCl, 30 mM EDTA, and 1% SDS). The digested samples were then precipitated with ethanol and resuspended in 5 µl Stop Solution (from Thermosequenase Cycle Sequencing Kit (USB): 95% formamide, 20 mM EDTA, 0.05% bromophenol blue, 0.05% xylene cyanol). Samples were heated at 80°C for 3 minutes, cooled on ice, and separated on 8% polyacrylamide/7 M urea sequencing gels. Dried gels were exposed using a phosphor screen (Bio-Rad) and bands were detected using a PhosphorImager (Molecular Dynamics). Sequencing ladders were prepared using Thermosequenase Cycle Sequencing Kit (USB).

### Construction of Lux-based Reporter Plasmids and Bioluminescence Measurements

Two reporter plasmids were constructed for each TFR of interest (Table S3). For the first, a DNA fragment containing the intergenic sequence between a TFR of interest and its divergent neighbor gene was cloned into pMU1* [33] in an orientation such that *lux* expression was driven by the promoter of the divergent neighbor (Figure 7A). The second construct had a DNA fragment containing the TFR gene as well as its intergenic sequence introduced to pMU1* in the same orientation as the first. In this construct, the TFR gene was transcribed by its natural promoter in the opposite direction to the *lux* operon (Figure 7A).

Host organisms for the reporters were designated by using protein BLAST to identify a streptomycete that does not possess any possible ortholog of the selected TFR (at least 40% identity in the amino acid sequence with at least 75% query coverage). 2×10^7^ colony forming units of the *Streptomyces* reporter spores were inoculated and grown for 16 hours to 20 hours. The overnight grown cells were then subcultured to set the starting OD (OD_450_ for *S. coelicolor* and OD_600_ for the other streptomycetes) at 0.05 (t = 0), and the cultures were measured for bioluminescence and OD every hour using VICTOR™ X Light 2030 luminescence reader (PerkinElmer) and Epoch microplate spectrophotometer (BioTek), respectively.

## Supporting Information

Figure S1 **SGR3979, SCO7222, SCO3367, SGR5269, and AtrA bind their intergenic DNAs, while SGR3402 does not.** (**A**) The indicated concentrations of SGR3979, SCO7222, SCO3367, or SGR5269 were incubated with a DNA fragment containing the entire sequence of the *SGR3978*/*SGR3979*, *SCO7222*/*SCO7223*, *SCO3366*/*SCO3367*, or *SGR5269*/*SGR5270* intergenic region. Unbound DNA is indicated by the bottom arrow (IGR), while the shifts representing protein-DNA complexes are indicated by the upper arrows. (**B**) Three probes for AtrA (IGR*_atrA_* → the 180 bp sequence from the *atA* translational start site; IGR*_SCO4119_* → the 180 bp sequence from the SCO4119 translational start site; IGR*_centre_* → the central 180 bp region between the *atrA* and *SCO4119* translational start sites) were prepared and incubated with the indicated concentrations of AtrA. (**C**) Four probes for SGR3402 (IGR*_SGR3403_*, 180 bp; IGR*_centre 1_*, 180 bp; IGR*_centre 2_*, 190 bp; and IGR*_SGR3402_*, 148 bp, partially cover the *SGR3402*/*SGR3403* intergenic regions in the order of the increasing distance to the *SGR3403* translational start site) were prepared and incubated with SGR3402.
(TIF)

Figure S2 **The interactions of SCO4099 and SGR3979 with their cognate intergenic sequences are specific.** (**A**) Gel mobility shift assays using 12.5 nM SCO4099. C (control), SCO4099 and labeled *SCO4098*/*SCO4099* intergenic probe; lanes 1 to 3, SCO4099 and labeled intergenic probe with 1x (lane 1), 10x (lane 2), or 100x (lane 3) unlabeled intergenic probe; lanes 4 to 6, SCO4099 and labeled intergenic probe with 1x (lane 4), 10x (lane 5), or 100x (lane 6) unlabeled non-specific control DNA (here, the intergenic sequence for SGR3979 was used due to its similar length to the SCO4099 intergenic sequence). (**B**) Gel mobility shift assays using 12.5 nM SGR3979. C (control), SGR3979 and labeled *SGR3978*/*SGR3979* intergenic probe; lanes 1 to 3, SGR3979 and labeled intergenic probe with 1x (lane 1), 10x (lane 2), or 100x (lane 3) unlabeled intergenic probe; lanes 4 to 6, SGR3979 and labeled intergenic probe with 1x (lane 4), 10x (lane 5), or 100x (lane 6) unlabeled non-specific control DNA (here, the intergenic sequence for SCO4099 was used).
(TIF)

Figure S3 **SGR5269, SCO3367, ActR, and SCO4099 show different protection patterns on their cognate intergenic sequences.** A DNA fragment containing the entire sequence of the *SGR5269*/*SGR5270*, *SCO3366*/*SCO3367*, *actR*/*actA*, or *SCO4098*/*SCO4099* intergenic region was exposed to DNase I in the presence of the indicated concentrations of the cognate TFR: (**A**) SGR5269, (**B**) SCO3367, (**C**) ActR, or (**D**) SCO4099. Two sequencing gels are shown for each TFR. For the left gel of each TFR, the primer that was extended toward the divergent neighboring gene was labeled at 5′-end to prepare the probe, while the other primer extended toward its own gene was labeled for the right gel. The regions protected by the TFRs are indicated by solid vertical lines. The numbers beside the lines indicate the start and end positions of the protected regions relative to the translational start site of the TFR-encoding gene.
(TIF)

Figure S4 **SGR3979, SCO3367, and SCO4099 repress their divergent targets, while SGR5269 and SGR3402 do not show any regulatory activity.** Compared to the cognate “Without TFR” constructs, (**A**) SGR3979, (**B**) SCO3367, and (**C**) SCO4099 had a negative effect on luminescence when expressed in *cis*, while (**D**) SGR5269 and (**E**) SGR3402 had no effect. Average bioluminescence values, measured in cps, as well as +/− standard deviation of the values were obtained from at least three independent readings.
(TIF)

Table S1 **Analysis of the TFRs having divergent neighbors.**
(PDF)

Table S2 **Types of protein products encoded by the divergent neighboring genes.**
(PDF)

Table S3 **Plasmids used in this work.**
(PDF)
