# Supplementary figures and images for: Genome Context as a Predictive Tool for Identifying Regulatory Targets of the TetR Family Transcriptional Regulators
Source: PLoS One. 2012 Nov 30;7(11):e50562. doi: 10.1371/journal.pone.0050562 (PMC3511530; doi:10.1371/journal.pone.0050562)

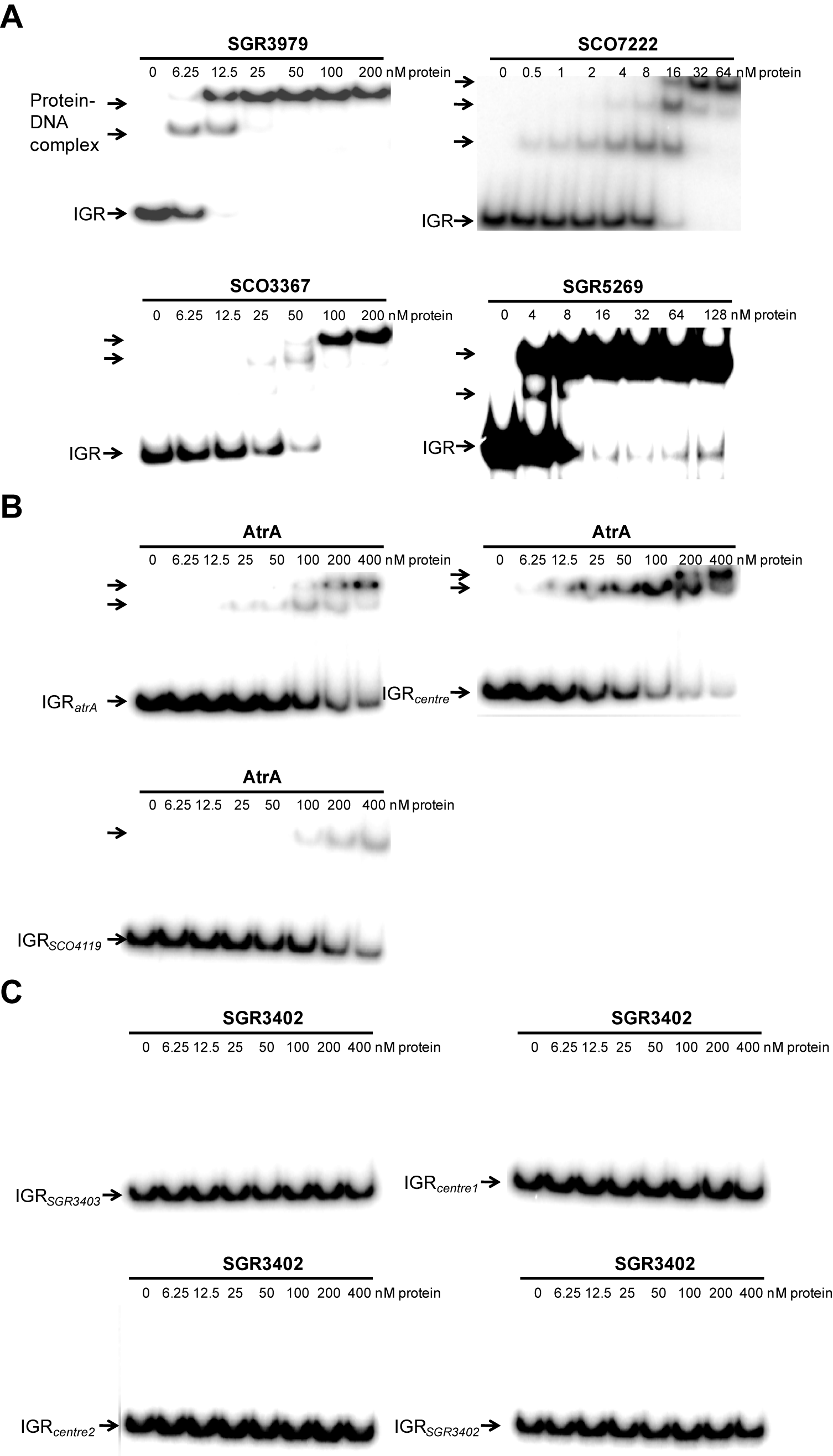

Supplement: Figure S1 — SGR3979, SCO7222, SCO3367, SGR5269, and AtrA bind their intergenic DNAs, while SGR3402 does not. (A) The indicated concentrations of SGR3979, SCO7222, SCO3367, or SGR5269 were incubated with a DNA fragment containing the entire sequence of the SGR3978/SGR3979, SCO7222/SCO7223, SCO3366/SCO3367, or SGR5269/SGR5270 intergenic region. Unbound DNA is indicated by the bottom arrow (IGR), while the shifts representing protein-DNA complexes are indicated by the upper arrows. (B) Three probes for AtrA (IGRatrA → the 180 bp sequence from the atA translational start site; IGRSCO4119 → the 180 bp sequence from the SCO4119 translational start site; IGRcentre → the central 180 bp region between the atrA and SCO4119 translational start sites) were prepared and incubated with the indicated concentrations of AtrA. (C) Four probes for SGR3402 (IGRSGR3403, 180 bp; IGRcentre 1, 180 bp; IGRcentre 2, 190 bp; and IGRSGR3402, 148 bp, partially cover the SGR3402/SGR3403 intergenic regions in the order of the increasing distance to the SGR3403 translational start site) were prepared and incubated with SGR3402. (TIF) [file pone.0050562.s001.tif]

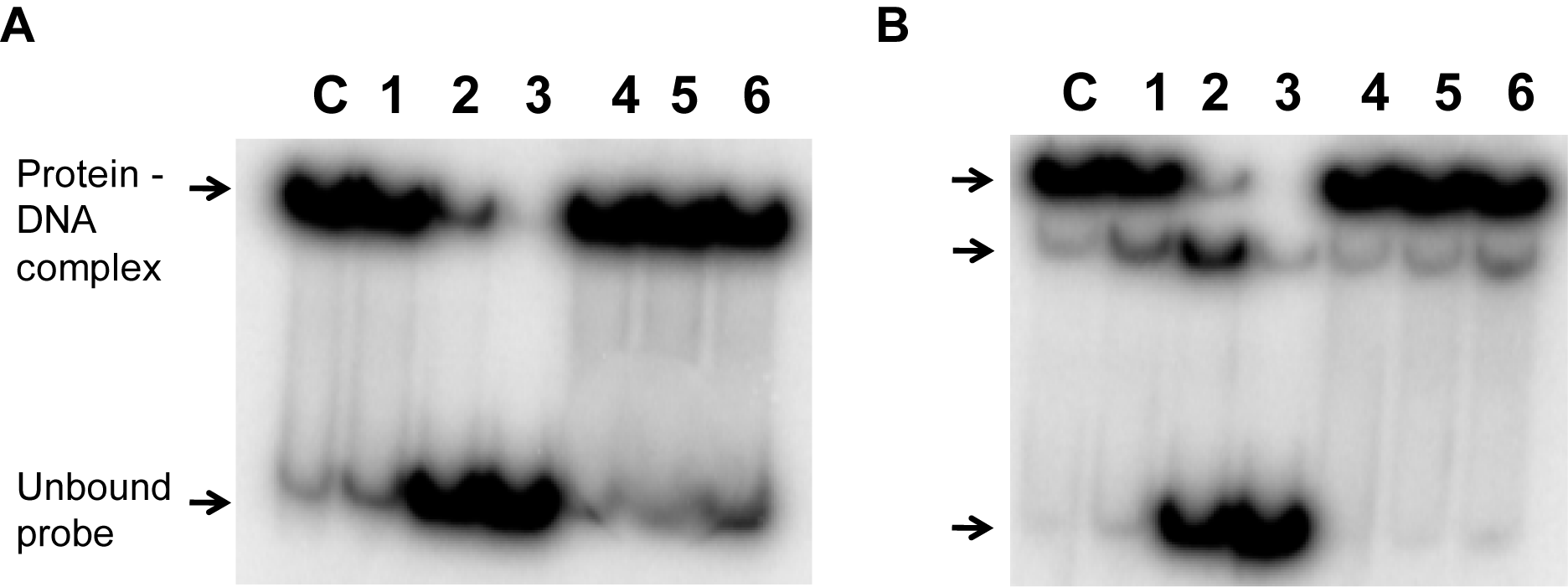

Supplement: Figure S2 — The interactions of SCO4099 and SGR3979 with their cognate intergenic sequences are specific. (A) Gel mobility shift assays using 12.5 nM SCO4099. C (control), SCO4099 and labeled SCO4098/SCO4099 intergenic probe; lanes 1 to 3, SCO4099 and labeled intergenic probe with 1x (lane 1), 10x (lane 2), or 100x (lane 3) unlabeled intergenic probe; lanes 4 to 6, SCO4099 and labeled intergenic probe with 1x (lane 4), 10x (lane 5), or 100x (lane 6) unlabeled non-specific control DNA (here, the intergenic sequence for SGR3979 was used due to its similar length to the SCO4099 intergenic sequence). (B) Gel mobility shift assays using 12.5 nM SGR3979. C (control), SGR3979 and labeled SGR3978/SGR3979 intergenic probe; lanes 1 to 3, SGR3979 and labeled intergenic probe with 1x (lane 1), 10x (lane 2), or 100x (lane 3) unlabeled intergenic probe; lanes 4 to 6, SGR3979 and labeled intergenic probe with 1x (lane 4), 10x (lane 5), or 100x (lane 6) unlabeled non-specific control DNA (here, the intergenic sequence for SCO4099 was used). (TIF) [file pone.0050562.s002.tif]

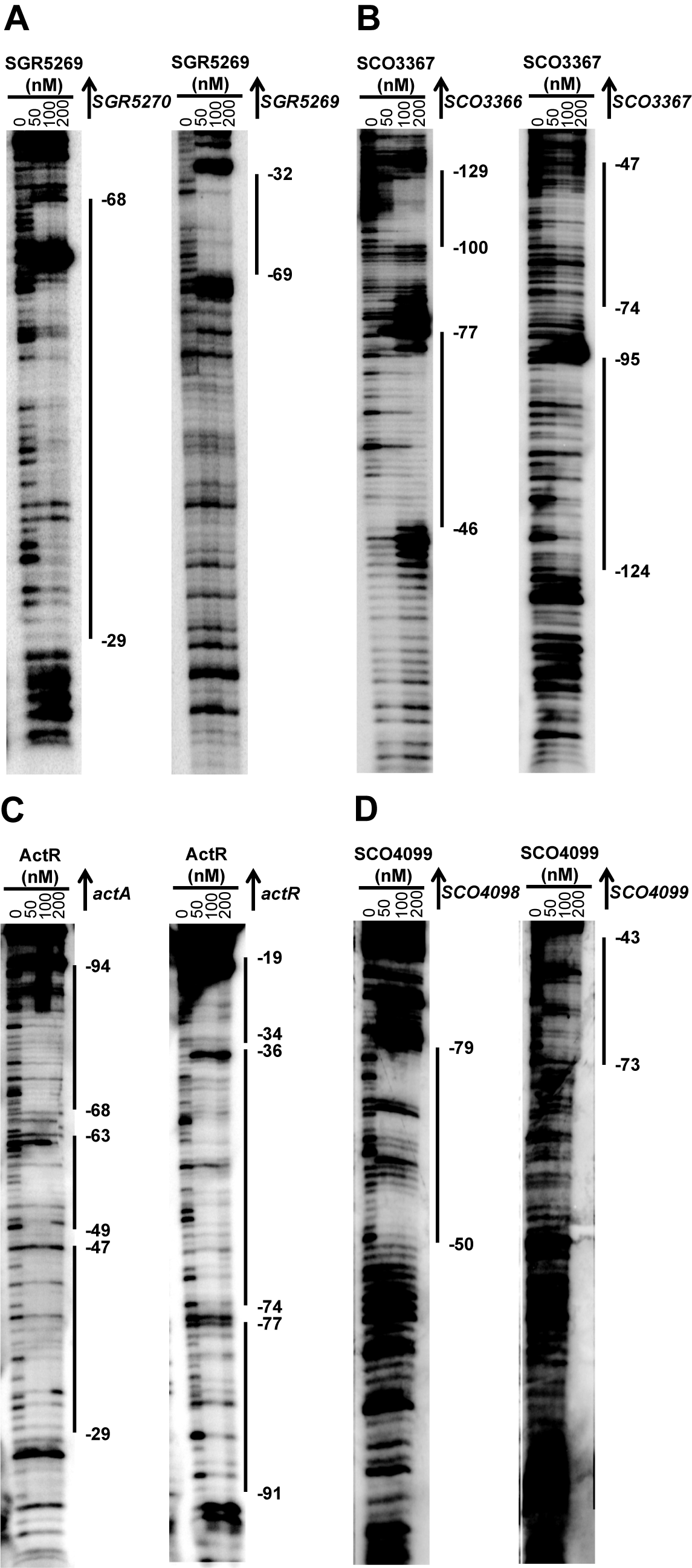

Supplement: Figure S3 — SGR5269, SCO3367, ActR, and SCO4099 show different protection patterns on their cognate intergenic sequences. A DNA fragment containing the entire sequence of the SGR5269/SGR5270, SCO3366/SCO3367, actR/actA, or SCO4098/SCO4099 intergenic region was exposed to DNase I in the presence of the indicated concentrations of the cognate TFR: (A) SGR5269, (B) SCO3367, (C) ActR, or (D) SCO4099. Two sequencing gels are shown for each TFR. For the left gel of each TFR, the primer that was extended toward the divergent neighboring gene was labeled at 5′-end to prepare the probe, while the other primer extended toward its own gene was labeled for the right gel. The regions protected by the TFRs are indicated by solid vertical lines. The numbers beside the lines indicate the start and end positions of the protected regions relative to the translational start site of the TFR-encoding gene. (TIF) [file pone.0050562.s003.tif]

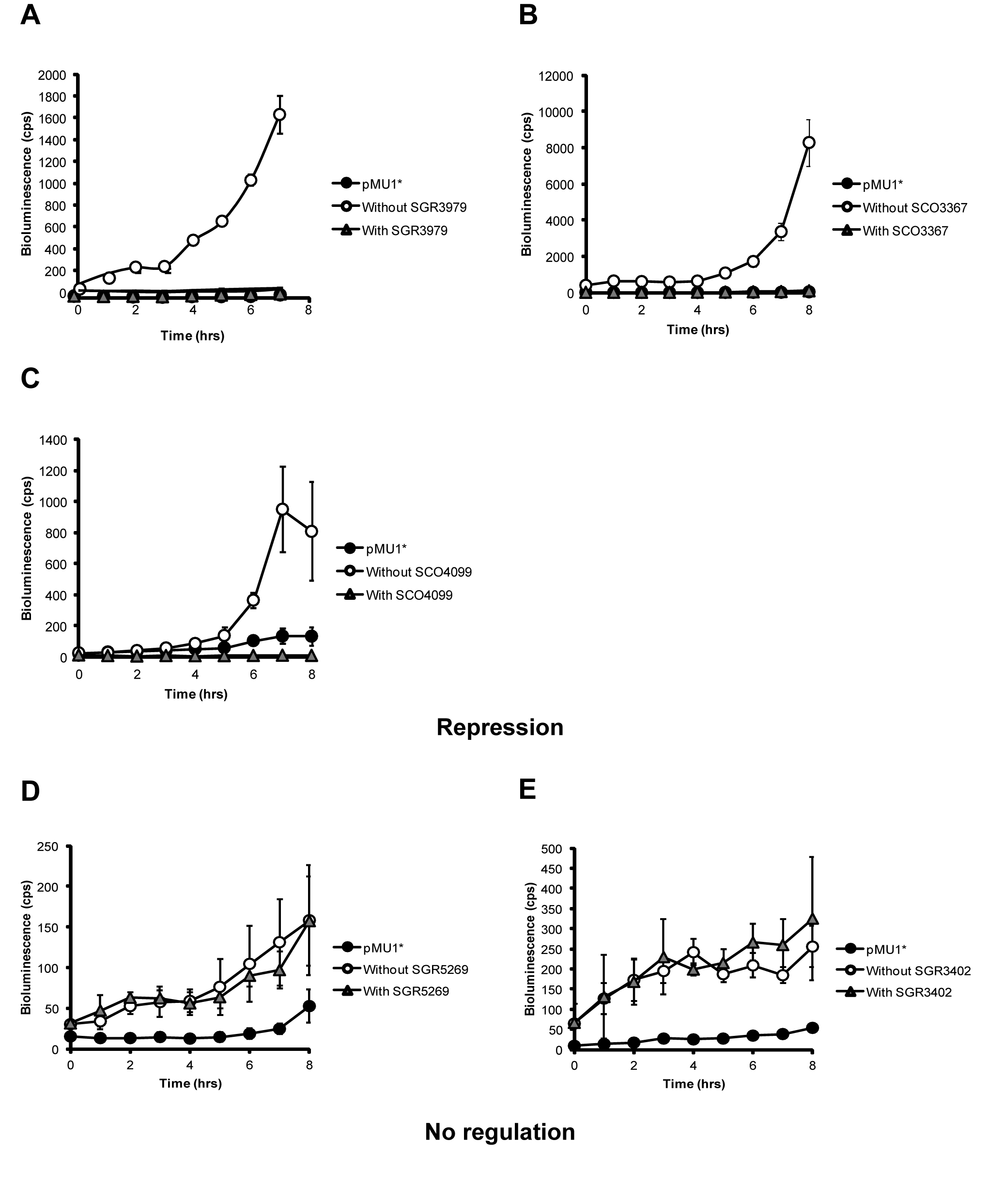

Supplement: Figure S4 — SGR3979, SCO3367, and SCO4099 repress their divergent targets, while SGR5269 and SGR3402 do not show any regulatory activity. Compared to the cognate “Without TFR” constructs, (A) SGR3979, (B) SCO3367, and (C) SCO4099 had a negative effect on luminescence when expressed in cis, while (D) SGR5269 and (E) SGR3402 had no effect. Average bioluminescence values, measured in cps, as well as +/− standard deviation of the values were obtained from at least three independent readings. (TIF) [file pone.0050562.s004.tif]
