# Supplementary material for: Genome Context as a Predictive Tool for Identifying Regulatory Targets of the TetR Family Transcriptional Regulators
Source: PLoS One. 2012 Nov 30;7(11):e50562. doi: 10.1371/journal.pone.0050562 (PMC3511530; doi:10.1371/journal.pone.0050562)
Supplement: Table S1 — Analysis of the TFRs having divergent neighbors. (PDF) [file pone.0050562.s005.pdf]

Table S1. Analysis of the TFRs having divergent neighbors

TFR	Length of intergenic DNA (bp)	Divergent neighbor	Putative divergent gene product[a]
TFRs with intergenic DNA <200 bp			
SCO0116	116	SCO0117	NADB_Rossmann superfamily (EC 1)
SCO0155	132	SCO0154	Thioredoxin_like superfamily (EC 1)
SCO0250	63	SCO0249	NADB_Rossmann superfamily (EC 1)
SCO0253	70	SCO0252	NADB_Rossmann superfamily (EC 1)
SCO0296	127	SCO0295	MFS transporter
SCO0310	108	SCO0311	AMP-binding superfamily (EC 6)
SCO0337	121	SCO0336	Saccharop_dh family (EC 1)
SCO0430	23	SCO0429	Aldo_ket_red superfamily (EC 1)
SCO0485	148	SCO0484	NADB_Rossmann superfamily (EC 1)
SCO0508	9	SCO0507	NADB_Rossmann superfamily (EC 1)
SCO0512	65	SCO0513	PerM family transporter
SCO0520	136	SCO0519	NADB_Rossmann superfamily (EC 1)
SCO0646	111	SCO0645	NADB_Rossmann superfamily (EC 1)
SCO0669	77	SCO0668	FAD_binding_4 superfamily (EC 1)
SCO0728	39	SCO0727	Protein of unassigned function
SCO0745	199	SCO0746	Protein of unassigned function
SCO0772	96	SCO0771	Protein of unassigned function
SCO0800	114	SCO0801	CypX superfamily (EC 1)
SCO0857	139	SCO0856	Membrane protein of unassigned function
SCO0887	139	SCO0888	FMN_red superfamily (EC 1)
SCO1003	98	SCO1002	NTF2_like superfamily (EC 5)
SCO1034	102	SCO1033	ABC-type transporter
SCO1135	188	SCO1134	fer2 superfamily (EC 1)
SCO1193	101	SCO1194	MFS transporter
SCO1210	170	SCO1209	ACAD superfamily (EC 1)
SCO1339	102	SCO1338	NADB_Rossmann superfamily (EC 1)
SCO1702	89	SCO1701	ACAD superfamily (EC 1)
SCO1718	63	SCO1719	ABC-type transporter
SCO2243	90	SCO2242	Membrane protein of unassigned function
SCO2319	114	SCO2318	Glycosyltransferase_GTB_type superfamily (EC 2)
SCO2374	115	SCO2373	MFS transporter
SCO2775	97	SCO2776	ACCA superfamily (EC 6)
SCO2815	33	SCO2814	AdoMet_Mtases superfamily (EC 2)
SCO2994	106	SCO2995	ABC-type transporter
SCO3167	148	SCO3168	Peptidase_S41 superfamily (EC 3)
SCO3315	128	SCO3314	NADB_Rossmann superfamily (EC 1)
SCO3367	158	SCO3366	MFS transporter
SCO3587	0	SCO3588	Erythro_esteras superfamily (EC 3)
SCO3769	75	SCO3770	CypX superfamily (EC 1)
SCO3979	80	SCO3978	NADB_Rossmann superfamily (EC 1)
SCO4008	136	SCO4007	MFS transporter
SCO4099	139	SCO4098	LbetaH superfamily (EC 2)
SCO4167	21	SCO4168	NADB_Rossmann superfamily (EC 1)
SCO4270	115	SCO4271	NADB_Rossmann superfamily (EC 1)
SCO4303	94	SCO4304	NADB_Rossmann superfamily (EC 1)
SCO4313	0	SCO4312	HDc superfamily (EC 3)
SCO4358	96	SCO4359	ABC-type transporter

SCO4421	134	SCO4422	Glo_EDI_BRP_like superfamily (EC 4)
SCO4450	69	SCO4449	SGL family (EC 4)
SCO4454	181	SCO4455	Membrane protein of unassigned function
SCO4461	125	SCO4462	MFS transporter
SCO4480	158	SCO4481	PKc_like superfamily (EC 2)
SCO4639	64	SCO4638	AdoMet_Mtases superfamily (EC 2)
SCO4871	72	SCO4870	NADB_Rossmann superfamily (EC 1)
SCO4898	109	SCO4899	Protein of unassigned function
SCO4940	101	SCO4939	Lactamase_B superfamily (EC 3)
SCO4942	120	SCO4943	Aldo_ket_red superfamily (EC 1)
SCO4952	144	SCO4951	Aldo_ket_red superfamily (EC 1)
SCO5068	155	SCO5069	NADB_Rossmann superfamily (EC 1)
SCO5082 (ActR)	110	SCO5083	MFS transporter
SCO5209	192	SCO5208	FIG superfamily (EC 3)
SCO5238	94	SCO5237	NADB_Rossmann superfamily (EC 1)
SCO5384	93	SCO5383	ABC-type transporter
SCO5483	127	SCO5484	Membrane protein of unassigned function
SCO5517	65	SCO5516	MFS transporter
SCO5532	91	SCO5531	DMT superfamily transporter
SCO5811	187	SCO5810	MFS transporter
SCO5906	153	SCO5905	RND superfamily transporter
SCO5951	147	SCO5950	MFS transporter
SCO5956	0	SCO5955	Protein of unassigned function
SCO6121	92	SCO6122	Membrane protein of unassigned function
SCO6144	151	SCO6145	PHP family (EC 3)
SCO6265 (ScbR)	117	SCO6266 (ScbA)	hot_dog superfamily (EC 4)
SCO6350	79	SCO6351	Abhydrolase_6 family (EC 3)
SCO6694	175	SCO6693	PAP2_like superfamily (EC 3)
SCO6792	74	SCO6791	ACAD superfamily (EC 1)
SCO7222	146	SCO7223	NADB_Rossmann superfamily (EC 1)
SCO7303	193	SCO7302	2OG-FeII_Oxy superfamily (EC 1)
SCO7364	105	SCO7365	Protein of unassigned function
SCO7441	86	SCO7440	Abhydrolase_6 family (EC 3)
SCO7552	99	SCO7553	NADB_Rossmann superfamily (EC 1)
SCO7602	127	SCO7601	Amidase superfamily (EC 3)
SCO7624	53	SCO7625	NADB_Rossmann superfamily (EC 1)
SCO7645	107	SCO7644	Saccharop_dh family (EC 1)
SCO7651	30	SCO7652	NAT_SF superfamily (EC 2)
SCO7694	62	SCO7693	NADB_Rossmann superfamily (EC 1)
SCO7719	54	SCO7720	Protein of unassigned function
SCO7794	67	SCO7793	Protein of unassigned function
SCO7809	51	SCO7810	NADB_Rossmann superfamily (EC 1)
SCO7815	13	SCO7814	NADB_Rossmann superfamily (EC 1)
SCO7824	0	SCO7825	Protein of unassigned function
SCP1.242 (MmfR)	194	SCP1.243 (MmfL)	hot_dog superfamily (EC 4)
SCP1.312	180	SCP1.311	NADB_Rossmann superfamily (EC 1)
SCP1.42	180	SCP1.43	NADB_Rossmann superfamily (EC 1)
SGR0049t	95	SGR0048t	NADB_Rossmann superfamily (EC 1)
SGR0138	77	SGR0137	Protein of unassigned function
SGR0218	71	SGR0219	NADB_Rossmann superfamily (EC 1)
SGR0266	84	SGR0265	Protein of unassigned function
SGR0372	61	SGR0371	Glyco_hydro_1 superfamily (EC 3)

SGR0429	114	SGR0428	Transcriptional regulator
SGR0829	159	SGR0828	PAP2_like superfamily (EC 3)
SGR0945	113	SGR0944	PNPOx_like superfamily (EC 1)
SGR1074	121	SGR1073	ABC-type transporter
SGR1207	133	SGR1208	AANH_like superfamily (EC 1)
SGR1523	108	SGR1524	Flavin_utilizing_monoxygenases superfamily (EC 1)
SGR1633	121	SGR1632	MFS transporter
SGR1965	138	SGR1964	NADB_Rossmann superfamily (EC 1)
SGR2032	64	SGR2031	Protein of unassigned function
SGR2153	90	SGR2154	ABC-type transporter
SGR2313	123	SGR2314	FIG superfamily (EC 3)
SGR2549	126	SGR2548	Siderophore interacting protein
SGR2601	104	SGR2602	Abhydrolase_6 family (EC 3)
SGR2777	127	SGR2776	MFS transporter
SGR2879	96	SGR2880	NADB_Rossmann superfamily (EC 1)
SGR3047	162	SGR3048	SRPBCC superfamily (EC 4)
SGR3098	104	SGR3099	NADB_Rossmann superfamily (EC 1)
SGR3119	162	SGR3118	ABC-type transporter
SGR3387	156	SGR3386	MFS transporter
SGR3746	156	SGR3745	ALDH-SF superfamily (EC 1)
SGR3979	144	SGR3978	ABC-type transporter
SGR4317	184	SGR4316	Peptidase_S41 superfamily (EC 3)
SGR4423	114	SGR4424	Protein of unassigned function
SGR4539	191	SGR4538	ABC-type transporter
SGR4760	123	SGR4759	ACCA superfamily (EC 6)
SGR4763	102	SGR4762	NADB_Rossmann superfamily (EC 1)
SGR4829	158	SGR4830	RND superfamily transporter
SGR5038	124	SGR5039	Protein of unassigned function
SGR5044	70	SGR5043	NADB_Rossmann superfamily (EC 1)
SGR5084	101	SGR5083	NADB_Rossmann superfamily (EC 1)
SGR5127	127	SGR5128	MFS transporter
SGR5186	182	SGR5187	Glycosyltransferase_GTB_type superfamily (EC 2)
SGR5798	90	SGR5799	ACAD superfamily (EC 1)
SGR5901	65	SGR5900	DMT superfamily transporter
SGR6082	74	SGR6081	RND superfamily transporter
SGR6196	83	SGR6197	SMR-type transporter
SGR6294	125	SGR6293	Membrane protein of unassigned function
SGR6310	187	SGR6311	ACAD superfamily (EC 1)
SGR6406	132	SGR6407	Protein of unassigned function
SGR6435	193	SGR6436	fer2 superfamily (EC 1)
SGR6536	150	SGR6535	Protein of unassigned function
SGR6627	106	SGR6626	NADB_Rossmann superfamily (EC 1)
SGR6910	114	SGR6911	RICIN superfamily (EC 3)
SGR6935	148	SGR6934	Saccharop_dh family (EC 1)
SGR7004	164	SGR7003	Aldo_ket_red superfamily (EC 1)
SGR7090t	95	SGR7091t	NADB_Rossmann superfamily (EC 1)
SAV0146	151	SAV0145	RND superfamily transporter
SAV0151	147	SAV0152	NADB_Rossmann superfamily (EC 1)
SAV0205	90	SAV0204	Membrane protein of unassigned function
SAV0448	122	SAV0449	Protein of unassigned function
SAV0488	119	SAV0489	Protein of unassigned function
SAV0675	126	SAV0676	NADB_Rossmann superfamily (EC 1)
SAV0754	106	SAV0753	Protein of unassigned function

SAV0775	170	SAV0774	NADB_Rossmann superfamily (EC 1)
SAV0880	181	SAV0881	NADB_Rossmann superfamily (EC 1)
SAV0882	59	SAV0883	NADB_Rossmann superfamily (EC 1)
SAV0913	117	SAV0912	NADB_Rossmann superfamily (EC 1)
SAV1068	169	SAV1067	NADB_Rossmann superfamily (EC 1)
SAV1218	62	SAV1217	Lactamase_B superfamily (EC 3)
SAV1359	83	SAV1358	NADB_Rossmann superfamily (EC 1)
SAV1439	148	SAV1438	Membrane protein of unassigned function
SAV1466	185	SAV1467	NADB_Rossmann superfamily (EC 1)
SAV1468	86	SAV1469	Protein of unassigned function
SAV1778	175	SAV1777	NADB_Rossmann superfamily (EC 1)
SAV1781	68	SAV1780	PNPOx_like superfamily (EC 1)
SAV2263	159	SAV2264	MFS transporter
SAV2268	68	SAV2267	ACAD superfamily (EC 1)
SAV2270	191	SAV2269 (AvaA)	hot_dog superfamily (EC 4)
SAV2381	153	SAV2380	ABC-type transporter
SAV2454	189	SAV2455	MFS transporter
SAV2727	184	SAV2728	MFS transporter
SAV2759	134	SAV2758	Protein of unassigned function
SAV2814	149	SAV2813	MFS transporter
SAV2871	80	SAV2872	ABC-type transporter
SAV3049	188	SAV3050	FIG superfamily (EC 3)
SAV3379	38	SAV3380	NADB_Rossmann superfamily (EC 1)
SAV3546	118	SAV3545	Protein of unassigned function
SAV3619	186	SAV3620	Peptidase_S41 superfamily (EC 3)
SAV3760	148	SAV3761	Protein of unassigned function
SAV3818	145	SAV3817	Glo_EDI_BRP_like superfamily (EC 4)
SAV4017	104	SAV4018	NADB_Rossmann superfamily (EC 1)
SAV4530	186	SAV4531	Esterase_lipase superfamily (EC 3)
SAV4701	189	SAV4702	MFS transporter
SAV4782	98	SAV4781	NADB_Rossmann superfamily (EC 1)
SAV5084	98	SAV5085	NADB_Rossmann superfamily (EC 1)
SAV5174	107	SAV5173	Abhydrolase_6 family (EC 3)
SAV5279	97	SAV5278	ACCA superfamily (EC 6)
SAV5333	167	SAV5334	RND superfamily transporter
SAV5724	181	SAV5725	Membrane protein of unassigned function
SAV5796	115	SAV5797	MFS transporter
SAV6352	137	SAV6351	MFS transporter
SAV6440	57	SAV6441	ADC superfamily (EC 4)
SAV6599	77	SAV6600	ACAD superfamily (EC 1)
SAV6701	185	SAV6700	Protein of unassigned function
SAV7046	113	SAV7045	FMN_red superfamily (EC 1)
SAV7127	120	SAV7128	ACAD superfamily (EC 1)
SAV7167	42	SAV7168	4Oxalocrotonate_Tautomerase superfamily (EC 5)
SAV7427	108	SAV7426	CypX superfamily (EC 1)
SAV7471	126	SAV7472	Protein of unassigned function
TFRs with intergenic DNA >200 bp			
SCO0241	345	SCO0240	NADB_Rossmann superfamily (EC 1)
SCO3207	238	SCO3208	Peptidase_M6 superfamily (EC 3)
SCO4118 (AtrA)	425	SCO4119	Pyr_redox superfamily (EC 1)
SCO5296	308	SCO5297	DUF772 superfamily (EC 2)

SCO5418	303	SCO5419	Thioredoxin_like superfamily (EC 1)
SCO6071 (CprB)	355	SCO6070	Periplasmic binding protein
SCO6286	237	SCO6287	Thioesterase family (EC 3)
SCO6323	385	SCO6324	HAD_like superfamily (EC 3)
SCO6775	232	SCO6776	Lactamase_B superfamily (EC 3)
SCO6784	245	SCO6785	CoA_transf_3 superfamily (EC 2)
SCO7347	236	SCO7346	MFS transporter
SGR1098	303	SGR1099	PAD_porph superfamily (EC 3)
SGR1284	241	SGR1283	DMT superfamily transporter
SGR2119	452	SGR2118	Thioredoxin_like superfamily (EC 1)
SGR3022	330	SGR3021	Glo_EDI_BRP_like superfamily (EC 4)
SGR3123	397	SGR3124	TauE/SafE exporter
SGR3127	228	SGR3126	Glyco_hydro_3 superfamily (EC 3)
SGR3402	601	SGR3403	MFS transporter
SGR3731 (ArpA)	1024	SGR3730	Protein of unassigned function
SGR3905 (AtrA-g)	565	SGR3906	Pyr_redox superfamily (EC 1)
SGR4211	251	SGR4210	Abhydrolase_5 family (EC 3)
SGR4271	292	SGR4270	Peptidase_M6 superfamily (EC 3)
SGR5223	281	SGR5222	MFS transporter
SGR5234	293	SGR5233	Amidinotransf superfamily (EC 2)
SGR5269	212	SGR5270	MFS transporter
SGR5284	692	SGR5285	Transcriptional regulator
SGR5789	222	SGR5788	Cupin_2 superfamily (EC 1)
SGR5970	323	SGR5969	P-loop_NTPase superfamily (EC 3)
SGR6382	683	SGR6383	Transcriptional regulator
SGR6383	683	SGR6382	Transcriptional regulator
SGR6390	368	SGR6389	MPP_superfamily (EC 3)
SGR6440	215	SGR6441	MFS transporter
SGR6912	280	SGR6911	RICIN superfamily (EC 3)
SGR6951	268	SGR6952	Protein of unassigned function
SAV0082	205	SAV0083	Protein of unassigned function
SAV0292	1123	SAV0291	rve superfamily (EC 2)
SAV0431	371	SAV0432	Membrane protein of unassigned function
SAV0508	386	SAV0507	NADB_Rossmann superfamily (EC 1)
SAV0566	245	SAV0565	Glo_EDI_BRP_like superfamily (EC 1)
SAV0576	899	SAV0575	CypX superfamily (EC 1)
SAV0585	265	SAV0586	PP2Cc superfamily (EC 3)
SAV0669	244	SAV0668	Glo_EDI_BRP_like superfamily (EC 4)
SAV1541	255	SAV1540	fer2 superfamily (EC 1)
SAV1711	231	SAV1712	PAP2_like superfamily (EC 3)
SAV2056	229	SAV2055	NADB_Rossmann superfamily (EC 1)
SAV2098	316	SAV2097	P-loop_NTPase superfamily (EC 3)
SAV2831	236	SAV2830	Thioredoxin_like superfamily (EC 1)
SAV3699	238	SAV3700	Peptidase_M6 superfamily (EC 3)
SAV4110	424	SAV4109	Pyr_redox superfamily (EC 1)
SAV5082	237	SAV5081	ABC-type transporter
SAV5854	208	SAV5855	Glycosyltransferase_GTB_type superfamily (EC 2)
SAV7510	209	SAV7509	Protein of unassigned function

[a] For the putative enzymes, their conserved domains identified by NCBI CD-Search as well as the predicted Enzyme Commission (EC) groups to which they potentially belong are indicated.
