# Supplementary material for: Genome Context as a Predictive Tool for Identifying Regulatory Targets of the TetR Family Transcriptional Regulators
Source: PLoS One. 2012 Nov 30;7(11):e50562. doi: 10.1371/journal.pone.0050562 (PMC3511530; doi:10.1371/journal.pone.0050562)
Supplement: Table S2 — Types of protein products encoded by the divergent neighboring genes. (PDF) [file pone.0050562.s006.pdf]

Table S2. Types of protein products encoded by the divergent neighboring genes

**Type of gene product**		**References**
EC 1: Oxidoreductases[a]		
Family of conserved domain	Number of TFRs	
NADB_Rossmann superfamily[b] (cl09931[c])	51	[1]
ACAD superfamily[b] (cl09933[c])	8	[2,3]
Aldo_ket_red superfamily[b] (cl00470[c])	4	[4]
CypX superfamily[b] (cl12078[c])	4	[5]
Thioredoxin_like superfamily[b] (cl00388[c])	4	[6]
Saccharop_dh (pfam03435)[d]	3	[7]
Pyr_redox superfamily[b] (cl15766)	3	[8]
fer2 superfamily[b] (cl00159) + Fer2_2 superfamily (cl08334[c])	3	[9]
FMN_red superfamily[b] (cl00438[c])	2	[10]
PNPOx_like superfamily[b] (cl00381[c])	2	[11]
Flavin_utilizing_monoxygenases superfamily[b] (cl07892[c])	1	[12]
ALDH-SF superfamily[b] (cl11961[c])	1	[13]
Cupin_2 superfamily[b] (cl09118[c])	1	[14]
FAD_binding_4 superfamily[b] (cl14794[c])	1	[15]
Glo_EDI_BRP_like superfamily[b] (cl14632[c])	1	[16]
2OG-FeII_Oxy superfamily[b] (cl15773[c])	1	[17]
AANH_like superfamily[b] (cl00292[c])	1	[18]
**Number of TFRs divergent to the EC 1 genes**	**91**	
EC 2: Transferases[a]		
Family of conserved domain	Number of TFRs	
Glycosyltransferase_GTB_type superfamily[b] (cl10013[c])	3	[19]
AdoMet_Mtases superfamily[b] (cl15754[c])	2	[20]
PKc_like superfamily[b] (cl09925[c])	1	[21]
CoA_transf_3 superfamily[b] (cl15643[c])	1	[22]
LbetaH superfamily[b] (cl00160[c])	1	[23,24]
NAT_SF superfamily[b] (cl00357[c])	1	[25]
Amidinotransf superfamily[b] (cl12043[c])	1	[26]
DUF772 superfamily[b] (cl15789[c])	1	[27]
rve superfamily[b] (cl01316[c])	1	[28]
**Number of TFRs divergent to the EC 2 genes**	**12**	
EC 3: Hydrolases[a]		
Family of conserved domain	Number of TFRs	
Abhydrolase_6 (pfam12697)[d]	4	[29]
Peptidase_S41 superfamily[b] (cl02526[c])	3	[30]
Peptidase_M6 superfamily[b] (cl11525[c])	3	[31]
Lactamase_B superfamily[b] (cl00446[c])	3	[32]
FIG superfamily[b] (cl00289[c])	3	[33]
PAP2_like superfamily[b] (cl00474[c])	3	[34]
P-loop_NTPase superfamily[b] (cl09099[c])	2	[35]
HAD_like superfamily[b] (cl11391[c])	1	[36]
Glyco_hydro_1 superfamily[b] (cl01046[c])	1	[37]
Erythro_esteras superfamily[b] (cl10069[c])	1	[38,39]
Glyco_hydro_3 superfamily[b] (cl07971[c])	1	[40]

MPP_superfamily[b] (cl13995[c])	1	[41]
RICIN superfamily[b] (cl15820[c])	1	[42]
Amidase superfamily[b] (cl11426[c])	1	[43]
PAD_porph superfamily[b] (cl01113[c])	1	[44]
Esterase_lipase superfamily[b] (cl12031[c])	1	[45]
PP2Cc superfamily[b] (cl00120[c])	1	[46]
Thioesterase (pfam00975)[d]	1	[47]
Abhydrolase_5 (pfam12695)[d]	1	[48]
HDc superfamily[b] (cl00076[c])	1	[49]
PHP (pfam02811)	1	[41]
**Number of TFRs divergent to the EC 3 genes**	**35**	
EC 4: Lyases[a]		
Family of conserved domain	Number of TFRs	
Glo_EDI_BRP_like superfamily[b] (cl14632[c])	4	[50]
hot_dog superfamily[b] (cl00509[c])	3	[51,52,53]
ADC superfamily[b] (cl01919[c])	1	[54]
SRPBCC superfamily[b] (cl14643[c])	1	[55]
SGL (pfam08450)[d]	1	[56]
**Number of TFRs divergent to the EC 4 genes**	**10**	
EC 5: Isomerases[a]		
Family of conserved domain	Number of TFRs	
4Oxalocrotonate_Tautomerase superfamily[b] (cl00235[c])	1	[57]
NTF2_like superfamily[b] (cl09109[c])	1	[58]
**Number of TFRs divergent to the EC 5 genes**	**2**	
EC 6: Ligases[a]		
Family of conserved domain	Number of TFRs	
ACCA superfamily[b] (cl15772[c])	3	[59]
AMP-binding superfamily[b] (cl15778[c])	1	[60]
**Number of TFRs divergent to the EC 6 genes**	**4**	
Membrane proteins		
Family of conserved domain	Number of TFRs	
Major facilitator superfamily (MFS) of transporters	26	[61]
ATP-binding cassette (ABC)-type transporters	14	[62]
Resistance-nodulation-division (RND) superfamily of transporters	5	[63]
Drug/Metabolite Transporter (DMT) superfamily of transporters	3	[64]
Small Multidrug Resistance (SMR)-type transporters	1	[65]
PerM family of transporters	1	[66]
TauE/SafE exporter	1	[67]
Other membrane proteins of unassigned function	10	
**Number of TFRs divergent to the genes encoding membrane proteins**	**61**	

	Number of TFRs
Proteins of other functions (e.g. transcription regulators)	**6**

	Number of TFRs
Proteins of unassigned functions	**29**

[a]The Enzyme Commission (EC) groups were used to classify putative enzymes according to the type of enzymatic reactions they were predicted to catalyze.
[b]The conserved domain superfamilies (as named in NCBI) were used to further group putative enzymes in each EC group.
[c]An accession number for a superfamily domain has the prefix “cl” for “cluster”.
[d]If a putative enzyme lacks a superfamily conserved domain, the protein family (pfam) motif was indicated instead.

References

1. Rossmann MG, Argos P (1976) Exploring structural homology of proteins. J Mol Biol 105: 75-95.
2. Kunau WH, Dommes V, Schulz H (1995) beta-oxidation of fatty acids in mitochondria, peroxisomes, and bacteria: a century of continued progress. Prog Lipid Res 34: 267-342.
3. Kim JJ, Battaile KP (2002) Burning fat: the structural basis of fatty acid beta-oxidation. Curr Opin Struct Biol 12: 721-728.
4. Sanli G, Dudley JI, Blaber M (2003) Structural biology of the aldo-keto reductase family of enzymes: catalysis and cofactor binding. Cell Biochem Biophys 38: 79-101.
5. Nelson DR, Kamataki T, Waxman DJ, Guengerich FP, Estabrook RW, et al. (1993) The P450 superfamily: update on new sequences, gene mapping, accession numbers, early trivial names of enzymes, and nomenclature. DNA Cell Biol 12: 1-51.
6. Loferer H, Wunderlich M, Hennecke H, Glockshuber R (1995) A bacterial thioredoxin-like protein that is exposed to the periplasm has redox properties comparable with those of cytoplasmic thioredoxins. J Biol Chem 270: 26178-26183.
7. Johansson E, Steffens JJ, Lindqvist Y, Schneider G (2000) Crystal structure of saccharopine reductase from *Magnaporthe grisea*, an enzyme of the alpha-aminoadipate pathway of lysine biosynthesis. Structure 8: 1037-1047.
8. Mande SS, Sarfaty S, Allen MD, Perham RN, Hol WG (1996) Protein-protein interactions in the pyruvate dehydrogenase multienzyme complex: dihydrolipoamide dehydrogenase complexed with the binding domain of dihydrolipoamide acetyltransferase. Structure 4: 277-286.
9. Romao MJ, Archer M, Moura I, Moura JJ, LeGall J, et al. (1995) Crystal structure of the xanthine oxidase-related aldehyde oxido-reductase from *D. gigas*. Science 270: 1170-1176.
10. van Der Ploeg JR, Iwanicka-Nowicka R, Bykowski T, Hryniewicz MM, Leisinger T (1999) The *Escherichia coli ssuEADCB* gene cluster is required for the utilization of sulfur from aliphatic sulfonates and is regulated by the transcriptional activator Cbl. J Biol Chem 274: 29358-29365.
11. Canaan S, Sulzenbacher G, Roig-Zamboni V, Scappuccini-Calvo L, Frassinetti F, et al. (2005) Crystal structure of the conserved hypothetical protein Rv1155 from *Mycobacterium tuberculosis*. FEBS Lett 579: 215-221.
12. Selengut JD, Haft DH (2010) Unexpected abundance of coenzyme F(420)-dependent enzymes in *Mycobacterium tuberculosis* and other actinobacteria. J Bacteriol 192: 5788-5798.
13. Kirch HH, Bartels D, Wei Y, Schnable PS, Wood AJ (2004) The ALDH gene superfamily of *Arabidopsis*. Trends Plant Sci 9: 371-377.
14. Titus GP, Mueller HA, Burgner J, Rodriguez De Cordoba S, Penalva MA, et al. (2000) Crystal structure of human homogentisate dioxygenase. Nat Struct Biol 7: 542-546.

15. Reed DW, Hartzell PL (1999) The *Archaeoglobus fulgidus* D-lactate dehydrogenase is a Zn(2+) flavoprotein. J Bacteriol 181: 7580-7587.
16. Que L, Jr., Widom J, Crawford RL (1981) 3,4-Dihydroxyphenylacetate 2,3-dioxygenase. A manganese(II) dioxygenase from *Bacillus brevis*. J Biol Chem 256: 10941-10944.
17. Holland PJ, Hollis T (2010) Structural and mutational analysis of *Escherichia coli* AlkB provides insight into substrate specificity and DNA damage searching. PLoS One 5: e8680.
18. Tsai MH, Saier MH, Jr. (1995) Phylogenetic characterization of the ubiquitous electron transfer flavoprotein families ETF-alpha and ETF-beta. Res Microbiol 146: 397-404.
19. Breton C, Snajdrova L, Jeanneau C, Koca J, Imberty A (2006) Structures and mechanisms of glycosyltransferases. Glycobiology 16: 29R-37R.
20. Lee PT, Hsu AY, Ha HT, Clarke CF (1997) A C-methyltransferase involved in both ubiquinone and menaquinone biosynthesis: isolation and identification of the *Escherichia coli ubiE* gene. J Bacteriol 179: 1748-1754.
21. Hanks SK, Quinn AM (1991) Protein kinase catalytic domain sequence database: identification of conserved features of primary structure and classification of family members. Methods Enzymol 200: 38-62.
22. Gruez A, Roig-Zamboni V, Valencia C, Campanacci V, Cambillau C (2003) The crystal structure of the *Escherichia coli* YfdW gene product reveals a new fold of two interlaced rings identifying a wide family of CoA transferases. J Biol Chem 278: 34582-34586.
23. Parisi G, Echave J (2004) The structurally constrained protein evolution model accounts for sequence patterns of the LbetaH superfamily. BMC Evol Biol 4: 41.
24. Beaman TW, Sugantino M, Roderick SL (1998) Structure of the hexapeptide xenobiotic acetyltransferase from *Pseudomonas aeruginosa*. Biochemistry 37: 6689-6696.
25. Isono K, Isono S (1980) Ribosomal protein modification in Escherichia coli. II. Studies of a mutant lacking the N-terminal acetylation of protein S18. Mol Gen Genet 177: 645-651.
26. Humm A, Fritsche E, Steinbacher S, Huber R (1997) Crystal structure and mechanism of human L-arginine:glycine amidinotransferase: a mitochondrial enzyme involved in creatine biosynthesis. EMBO J 16: 3373-3385.
27. Davies DR, Mahnke Braam L, Reznikoff WS, Rayment I (1999) The three-dimensional structure of a Tn5 transposase-related protein determined to 2.9-A resolution. J Biol Chem 274: 11904-11913.
28. Dyda F, Hickman AB, Jenkins TM, Engelman A, Craigie R, et al. (1994) Crystal structure of the catalytic domain of HIV-1 integrase: similarity to other polynucleotidyl transferases. Science 266: 1981-1986.
29. Ollis DL, Cheah E, Cygler M, Dijkstra B, Frolow F, et al. (1992) The alpha/beta hydrolase fold. Protein Eng 5: 197-211.
30. Pallen MJ, Lam AC, Loman N (2001) Tricorn-like proteases in bacteria. Trends Microbiol 9: 518-521.
31. Lovgren A, Zhang M, Engstrom A, Dalhammar G, Landen R (1990) Molecular characterization of immune inhibitor A, a secreted virulence protease from *Bacillus thuringiensis*. Mol Microbiol 4: 2137-2146.
32. Ullah JH, Walsh TR, Taylor IA, Emery DC, Verma CS, et al. (1998) The crystal structure of the L1 metallo-beta-lactamase from *Stenotrophomonas maltophilia* at 1.7 A resolution. J Mol Biol 284: 125-136.
33. Chen L, Roberts MF (2000) Overexpression, purification, and analysis of complementation behavior of *E. coli* SuhB protein: comparison with bacterial and archaeal inositol monophosphatases. Biochemistry 39: 4145-4153.
34. Stukey J, Carman GM (1997) Identification of a novel phosphatase sequence motif. Protein Sci 6: 469-472.
35. Korolev S, Hsieh J, Gauss GH, Lohman TM, Waksman G (1997) Major domain swiveling revealed by the crystal structures of complexes of *E. coli* Rep helicase bound to single-stranded DNA and ADP. Cell 90: 635-647.
36. Hisano T, Hata Y, Fujii T, Liu JQ, Kurihara T, et al. (1996) Crystal structure of L-2-haloacid dehalogenase from *Pseudomonas* sp. YL. An alpha/beta hydrolase structure that is different from the alpha/beta hydrolase fold. J Biol Chem 271: 20322-20330.
37. Old LA, Lowes S, Russell RR (2006) Genomic variation in *Streptococcus mutans*: deletions affecting the multiple pathways of beta-glucoside metabolism. Oral Microbiol Immunol 21: 21-27.

38. Ounissi H, Courvalin P (1985) Nucleotide sequence of the gene *ereA* encoding the erythromycin esterase in *Escherichia coli*. Gene 35: 271-278.
39. Arthur M, Autissier D, Courvalin P (1986) Analysis of the nucleotide sequence of the *ereB* gene encoding the erythromycin esterase type II. Nucleic Acids Res 14: 4987-4999.
40. Varghese JN, Hrmova M, Fincher GB (1999) Three-dimensional structure of a barley beta-D-glucan exohydrolase, a family 3 glycosyl hydrolase. Structure 7: 179-190.
41. Aravind L, Koonin EV (1998) Phosphoesterase domains associated with DNA polymerases of diverse origins. Nucleic Acids Res 26: 3746-3752.
42. Robertus J (1991) The structure and action of ricin, a cytotoxic N-glycosidase. Semin Cell Biol 2: 23-30.
43. Chang TH, Abelson J (1990) Identification of a putative amidase gene in yeast *Saccharomyces cerevisiae*. Nucleic Acids Res 18: 7180.
44. Shirai H, Blundell TL, Mizuguchi K (2001) A novel superfamily of enzymes that catalyze the modification of guanidino groups. Trends Biochem Sci 26: 465-468.
45. Hemila H, Koivula TT, Palva I (1994) Hormone-sensitive lipase is closely related to several bacterial proteins, and distantly related to acetylcholinesterase and lipoprotein lipase: identification of a superfamily of esterases and lipases. Biochim Biophys Acta 1210: 249-253.
46. Barak I, Behari J, Olmedo G, Guzman P, Brown DP, et al. (1996) Structure and function of the *Bacillus* SpoIIE protein and its localization to sites of sporulation septum assembly. Mol Microbiol 19: 1047-1060.
47. Schneider A, Marahiel MA (1998) Genetic evidence for a role of thioesterase domains, integrated in or associated with peptide synthetases, in non-ribosomal peptide biosynthesis in *Bacillus subtilis*. Arch Microbiol 169: 404-410.
48. Derewenda ZS, Derewenda U (1998) The structure and function of platelet-activating factor acetylhydrolases. Cell Mol Life Sci 54: 446-455.
49. Aravind L, Koonin EV (1998) The HD domain defines a new superfamily of metal-dependent phosphohydrolases. Trends Biochem Sci 23: 469-472.
50. Thornalley PJ (2003) Glyoxalase I--structure, function and a critical role in the enzymatic defence against glycation. Biochem Soc Trans 31: 1343-1348.
51. Takano E, Chakraburtty R, Nihira T, Yamada Y, Bibb MJ (2001) A complex role for the gamma-butyrolactone SCB1 in regulating antibiotic production in *Streptomyces coelicolor* A3(2). Mol Microbiol 41: 1015-1028.
52. Yeats C, Bentley S, Bateman A (2003) New knowledge from old: *in silico* discovery of novel protein domains in *Streptomyces coelicolor*. BMC Microbiol 3: 3.
53. Horinouchi S, Kumada Y, Beppu T (1984) Unstable genetic determinant of A-factor biosynthesis in streptomycin-producing organisms: cloning and characterization. J Bacteriol 158: 481-487.
54. Gerischer U, Durre P (1990) Cloning, sequencing, and molecular analysis of the acetoacetate decarboxylase gene region from *Clostridium acetobutylicum*. J Bacteriol 172: 6907-6918.
55. Iyer LM, Koonin EV, Aravind L (2001) Adaptations of the helix-grip fold for ligand binding and catalysis in the START domain superfamily. Proteins 43: 134-144.
56. Mukhtar TA, Koteva KP, Hughes DW, Wright GD (2001) Vgb from *Staphylococcus aureus* inactivates streptogramin B antibiotics by an elimination mechanism not hydrolysis. Biochemistry 40: 8877-8886.
57. Roper DI, Subramanya HS, Shingler V, Wigley DB (1994) Preliminary crystallographic analysis of 4-oxalocrotonate tautomerase reveals the oligomeric structure of the enzyme. J Mol Biol 243: 799-801.
58. Ha NC, Choi G, Choi KY, Oh BH (2001) Structure and enzymology of Delta5-3-ketosteroid isomerase. Curr Opin Struct Biol 11: 674-678.
59. Marini P, Li SJ, Gardiol D, Cronan JE, Jr., de Mendoza D (1995) The genes encoding the biotin carboxyl carrier protein and biotin carboxylase subunits of *Bacillus subtilis* acetyl coenzyme A carboxylase, the first enzyme of fatty acid synthesis. J Bacteriol 177: 7003-7006.
60. Conti E, Franks NP, Brick P (1996) Crystal structure of firefly luciferase throws light on a superfamily of adenylate-forming enzymes. Structure 4: 287-298.
61. Saidijam M, Benedetti G, Ren Q, Xu Z, Hoyle CJ, et al. (2006) Microbial drug efflux proteins of the major facilitator superfamily. Curr Drug Targets 7: 793-811.

62. Tomii K, Kanehisa M (1998) A comparative analysis of ABC transporters in complete microbial genomes. Genome Res 8: 1048-1059.
63. Tseng TT, Gratwick KS, Kollman J, Park D, Nies DH, et al. (1999) The RND permease superfamily: an ancient, ubiquitous and diverse family that includes human disease and development proteins. J Mol Microbiol Biotechnol 1: 107-125.
64. Tate CG, Muiry JA, Henderson PJ (1992) Mapping, cloning, expression, and sequencing of the *rhaT* gene, which encodes a novel L-rhamnose-H^+ transport protein in *Salmonella typhimurium* and *Escherichia coli*. J Biol Chem 267: 6923-6932.
65. Yerushalmi H, Lebendiker M, Schuldiner S (1995) EmrE, an *Escherichia coli* 12-kDa multidrug transporter, exchanges toxic cations and H^+ and is soluble in organic solvents. J Biol Chem 270: 6856-6863.
66. Ravcheev DA, Gel'fand MS, Mironov AA, Rakhmaninova AB (2002) [Purine regulon of gamma-proteobacteria: a detailed description]. Genetika 38: 1203-1214.
67. Weinitschke S, Denger K, Cook AM, Smits TH (2007) The DUF81 protein TauE in *Cupriavidus necator* H16, a sulfite exporter in the metabolism of C2 sulfonates. Microbiology 153: 3055-3060.
