# Supplementary material for: Genome Context as a Predictive Tool for Identifying Regulatory Targets of the TetR Family Transcriptional Regulators
Source: PLoS One. 2012 Nov 30;7(11):e50562. doi: 10.1371/journal.pone.0050562 (PMC3511530; doi:10.1371/journal.pone.0050562)
Supplement: Table S3 — Plasmids used in this work. (PDF) [file pone.0050562.s007.pdf]

Table S3. Plasmids used in this work

Plasmid	Description (selection marker)[a]	Background	Reference
pET28a	Vector for His_6-tagged protein overexpression (Kan^r)		Novagen
pET28a-ActR	ActR-overexpressing vector for protein purification (Kan^r)	pET28a	[1]
pTO7222	SCO7222-overexpressing vector for protein purification (Kan^r)	pET28a	[2]
pET28a-SCO3367	SCO3367-overexpressing vector for protein purification (Kan^r)	pET28a	This work
pET28a-SCO4099	SCO4099-overexpressing vector for protein purification (Kan^r)	pET28a	This work
pET28a-AtrA	AtrA-overexpressing vector for protein purification (Kan^r)	pET28a	This work
pET28a-SGR3979	SGR3979-overexpressing vector for protein purification (Kan^r)	pET28a	This work
pET28a-SGR5269	SGR5269-overexpressing vector for protein purification (Kan^r)	pET28a	This work
pET28a-SGR6912	SGR6912-overexpressing vector for protein purification (Kan^r)	pET28a	This work
pET28a-SGR3402	SGR3402-overexpressing vector for protein purification (Kan^r)	pET28a	This work
pMU1*	Promoterless *luxCDABE* operon, flanked by transcriptional terminators, preceded by in-frame stop codon and ribosome binding site, *aac(3)IV* ($Apra^r$)	pRT801	[3]
pMU1*-IGR(ActR)	The *actR/actA* intergenic region fused to *luxCDABE* ($Apra^r$)	pMU1*	This work
pMU1*-ActR-IGR	The *actR* gene and the intergenic region fused to *luxCDABE* ($Apra^r$)	pMU1*	This work
pMU1*-IGR(SCO7222)	The *SCO7222/SCO7223* intergenic region fused to *luxCDABE* ($Apra^r$)	pMU1*	This work
pMU1*-SCO7222-IGR	The *SCO7222* gene and the intergenic region fused to *luxCDABE* ($Apra^r$)	pMU1*	This work
pMU1*-IGR(SCO3367)	The *SCO336/SCO3367* intergenic region fused to *luxCDABE* ($Apra^r$)	pMU1*	This work
pMU1*-SCO3367-IGR	The *SCO3367* gene and the intergenic region fused to *luxCDABE* ($Apra^r$)	pMU1*	This work
pMU1*-IGR(SCO4099)	The *SCO4098/SCO4099* intergenic region fused to *luxCDABE* ($Apra^r$)	pMU1*	This work
pMU1*-SCO4099-IGR	The *SCO4099* gene and the intergenic region fused to *luxCDABE* ($Apra^r$)	pMU1*	This work
pMU1*-IGR(AtrA)	The *atrA/SCO4119* intergenic region fused to *luxCDABE* ($Apra^r$)	pMU1*	This work
pMU1*-AtrA-IGR	The *atrA* gene and the intergenic region fused to *luxCDABE* ($Apra^r$)	pMU1*	This work
pMU1*-IGR(SGR3979)	The *SGR3978/SGR3979* intergenic region fused to *luxCDABE* ($Apra^r$)	pMU1*	This work
pMU1*-SGR3979-IGR	The *SGR3979* gene and the intergenic region fused to *luxCDABE* ($Apra^r$)	pMU1*	This work
pMU1*-IGR(SGR5269)	The *SGR5269/SGR5270* intergenic region fused to *luxCDABE* ($Apra^r$)	pMU1*	This work
pMU1*-	The *SGR5269* gene and the intergenic	pMU1*	This work

SGR5269-IGR	region fused to *luxCDABE* (Apra^r)		
pMU1*-IGR(SGR6912)	The *SGR6911/SGR6912* intergenic region fused to *luxCDABE* (Apra^r)	pMU1*	This work
pMU1*-SGR6912-IGR	The *SGR6912* gene and the intergenic region fused to *luxCDABE* (Apra^r)	pMU1*	This work
pMU1*-IGR(SGR3402)	The *SGR3402/SGR3403* intergenic region fused to *luxCDABE* (Apra^r)	pMU1*	This work
pMU1*-SGR3402-IGR	The *SGR3402* gene and the intergenic region fused to *luxCDABE* (Apra^r)	pMU1*	This work

[a] Kan, kanamycin resistance and Apra, apramycin resistance

References

1. Tahlan K, Ahn SK, Sing A, Bodnaruk TD, Willems AR, et al. (2007) Initiation of actinorhodin export in *Streptomyces coelicolor*. Mol Microbiol 63: 951-961.
2. Ahn SK, Tahlan K, Yu Z, Nodwell J (2007) Investigation of transcription repression and small-molecule responsiveness by TetR-like transcription factors using a heterologous *Escherichia coli*-based assay. J Bacteriol 189: 6655-6664.
3. Craney A, Hohenauer T, Xu Y, Navani NK, Li Y, et al. (2007) A synthetic *luxCDABE* gene cluster optimized for expression in high-GC bacteria. Nucleic Acids Res 35: e46.
